# Sex steroid hormones: an overlooked yet fundamental factor in oral homeostasis in humans

**DOI:** 10.3389/fendo.2024.1400640

**Published:** 2024-07-23

**Authors:** Pilar E. Cornejo Ulloa, B. P. Krom, Linda J. Schoonmade, M. H. van der Veen

**Affiliations:** ^1^ Department of Preventive Dentistry, Academic Centre for Dentistry Amsterdam (ACTA), Amsterdam, Netherlands; ^2^ Medical Library, Amsterdam UMC, location VUmc, VU Amsterdam, Amsterdam, Netherlands; ^3^ Department of Paediatric Dentistry, Academic Centre for Dentistry Amsterdam (ACTA), Amsterdam, Netherlands

**Keywords:** sex steroid hormones, oral cavity, oral endocrinology, sex steroid hormone receptors, oral homeostasis, humans

## Abstract

Sex steroid hormones (SSH) are extremely versatile molecules with a myriad of physiological functions. Next to their well-known role in sexual development and reproduction, SSH play active roles in practically every tissue in the human body, including the oral cavity. It has long been demonstrated that periodontal tissues express SSH receptors and therefore are responsive to the presence of SSH. Interestingly, SSH not only interact with the periodontal tissues but also with other tissues in the oral cavity such as dental enamel, pulp, cementum, oral mucosa, and salivary glands. Questions concerning the possible physiological functions of these receptors and their role in maintenance of oral health, remain unanswered. The purpose of this scoping review was to gather and summarize all the available evidence on the role of SSH in physiological processes in the oral cavity in humans. Two comprehensive literature searches were performed. References were screened and selected based on title, abstract and full text according to our inclusion criteria. Both searches yielded 18,992 results of which 73 were included. Results were divided into four categories: (1) Periodontium; (2) Dental structure; (3) Mucosa; and (4) Salivary glands. The interaction of these tissues with progestagens, androgens and estrogens are summarized. Sex steroid hormones are an overlooked yet fundamental factor in oral homeostasis. They play important roles in the development and function of the periodontium, dental structure, mucosa and salivary glands. Dentists and healthcare providers should consider these hormonal factors when assessing and treating oral health conditions.

## Graphical Abstract

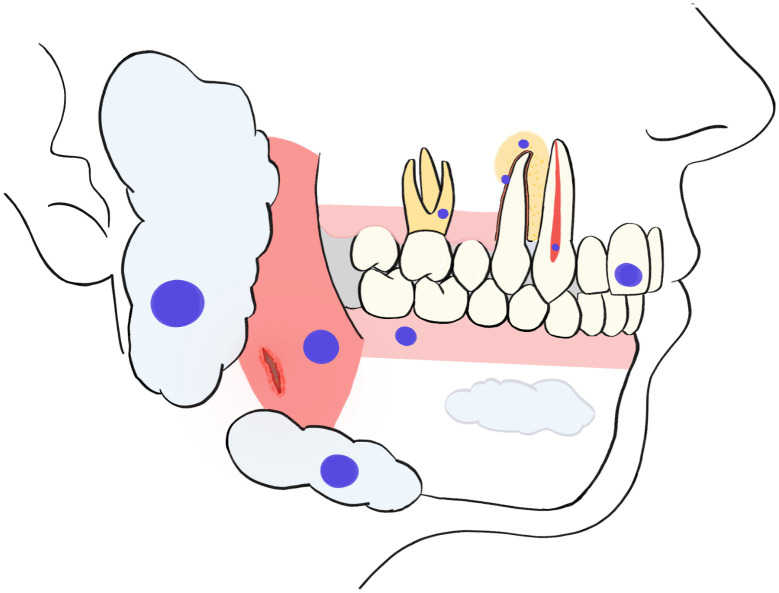

## 1 Introduction

When asked about the role of sex steroid hormones (SSH) on the human body, the first thought that comes to mind is their essential and primary function on the sexual and reproductive system. Although this holds true and is indeed the most studied and well-known role of SSH, it is by no means their only known function. These hormones are incredibly versatile and capable of inducing various physiological responses (1). Alongside their primary and well-known role in the sexual and reproductive system, SSH are involved in a variety of biological processes that bear no clear relationship with their main function. SSH –namely progestagens, androgens and estrogens– are known to act as appetite modulators (2), to influence skeletal muscle strength and power (3), to play a role in adipose tissue regulation (4, 5), bone mineral density (6) and regulation of the immune response (7), to name a few. Considering SSH’s versatility and ubiquity, it is not surprising that these molecules also play a role in the oral cavity (8).

**Figure 1:**
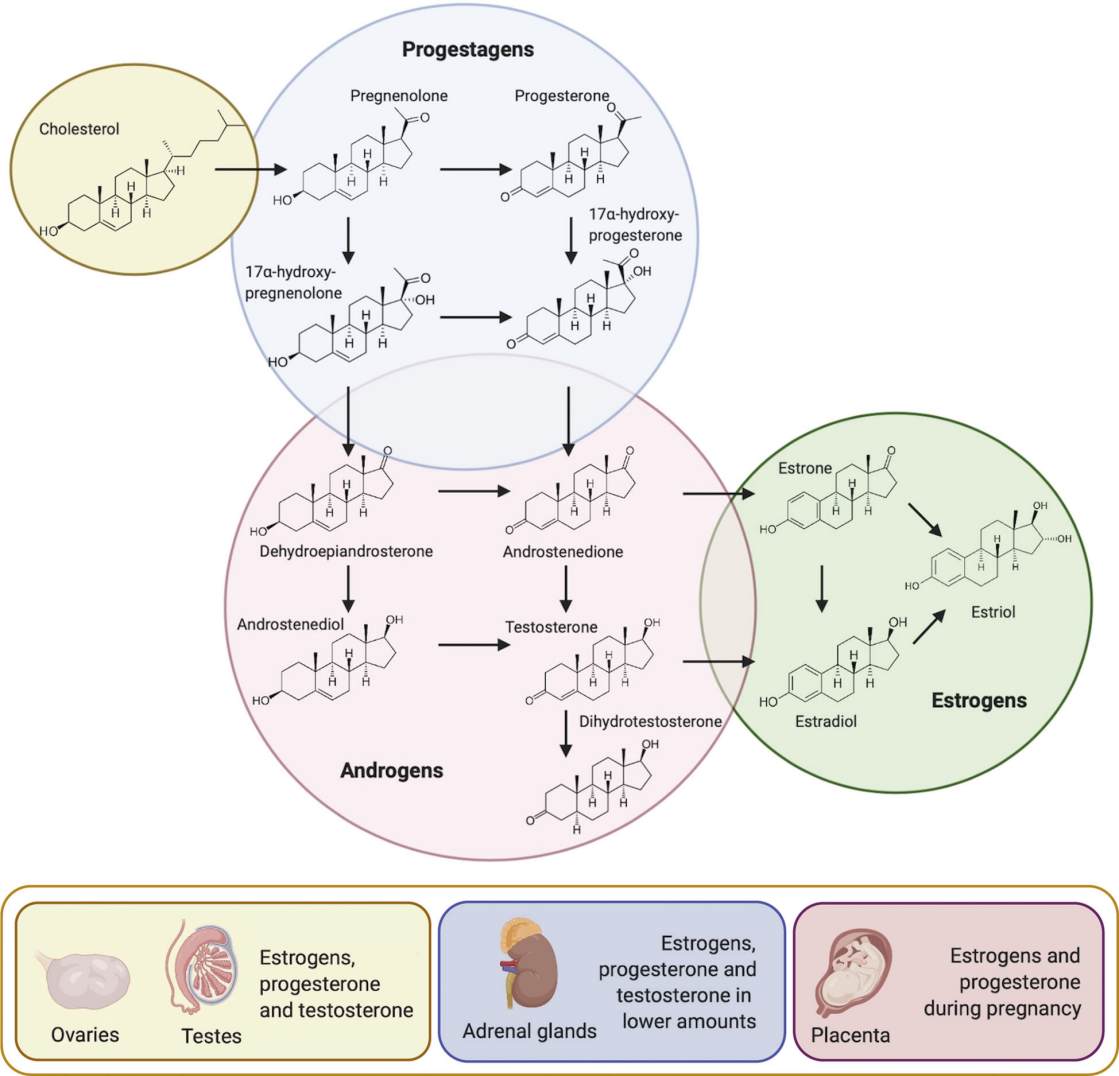
Steroidogenesis and main sources of sex steroid hormones. Cholesterol acts as the precursor molecule for the synthesis of the three classes of sex steroid hormones: progestagens, androgens and estrogens. Once P450_SCC_ has converted cholesterol into pregnenolone in the inner membrane of the mitochondria, pregnenolone passively diffuses to the smooth endoplasmic reticulum for further conversion. Reproductive organs are the main source of sex steroid hormones, followed by the adrenal cortex and during pregnancy, by the placenta (Modified from (9).

Synthesis of SSH begins with the enzymatic process known as steroidogenesis (**Figure 1**), where their precursor –cholesterol– is converted into biologically active SSH (10). Cholesterol is transported to the inner membrane of the mitochondria by specific proteins. The steroidogenic acute regulatory protein (StAR), and translocator protein (TSPO), in a complex with proteins such as voltage-dependent anion channel (VDAC), ATPase family AAA-domain containing protein 3A (ATAD3A), amongst others, are involved in this process. Once cholesterol has reached the inner membrane of the mitochondria, it is further converted into pregnenolone by the cholesterol side-chain cleavage enzyme (P450_SCC_; CYP11A1) (10, 11). Pregnenolone will then passively diffuse from the mitochondria to the smooth endoplasmic reticulum for further conversion into either progesterone by 3-beta-hydroxysteroid dehydrogenase (3β-HSD) or 17-alpha-hydroxypregnenolone by 17-alpha-hydroxylase/17,20-lyase (CYP17) (12). CYP17 and 3β-HSD can respectively convert progesterone and 17-alpha-hydroxypregnenolone into 17-alpha-hydroxyprogesterone. Pregnenolone, progesterone, 17-alpha-hydroxypregnenolone and 17-alpha-hydroxyprogesterone compose the progestagens class. CYP17 can go on to further convert 17-alpha-hydroxypregnenolone into dehydroepiandrosterone (DHEA) and 17-alpha-hydroxyprogesterone into androstenedione. DHEA will then be the precursor of androstenedione, androstenediol, testosterone, and dihydrotestosterone (DHT). These four SSH, together with DHEA compose the androgens class. Aromatization of androstenedione and testosterone will originate estrone and estradiol, respectively. Together with estriol, these three SSH compose the estrogens class (10).

In mammals, these molecules are mainly secreted by the testis, ovaries, adrenal cortex and during pregnancy, by the placenta (**Figure 1**) (13). Interestingly, the biosynthesis of sex steroid hormones has also been observed in the central nervous system, skin, and adipose tissue, albeit at a lower rate than in the reproductive organs (14–18).

Classical delivery modes of SSH are endocrine, paracrine, and autocrine. The endocrine mode involves hormone secretion and transport via blood vessels to reach distant target tissues. Paracrine delivery involves local synthesis of SSH and diffusion through extracellular fluid, covering smaller distances, usually within the same organ. In the case of the autocrine mode of delivery and action, a cell is activated by its own hormonal signals, becoming thus both hormone source and target (19).

Once SSH are synthesized and delivered, a high percentage binds to plasma proteins (such as SHBG). The remaining non-bound fraction of the hormones can bind to specific intracellular, membrane-associated, or transmembrane receptors, activating a signaling cascade that results in biological effects (20, 21).

It has long been demonstrated that periodontal tissues exhibit SSH receptors and therefore are susceptible to the presence of SSH (22–24). However, questions such as the possible physiological functions of these receptors or how they engage in the maintenance of oral health, remain unanswered.

The purpose of this review is to gather and summarize all the available evidence on the role of SSH in normal physiological processes in the oral cavity in humans. This includes how SSH influence oral homeostasis and what is known about their role on the development and function of different oral tissues. Clinical implications and future challenges will be discussed.

## 2 Materials and methods

### 2.1 Search and protocol

This scoping review was conducted in accordance with the Preferred for Systematic reviews and Meta-Analyses extension for Scoping Reviews (PRISMA-Scr) checklist explanation (25). In collaboration with a medical librarian (LS), two comprehensive searches were carried out on different dates. The first search, from inception to April 6^th^, 2020 was carried out in the bibliographic database PubMed. The second search –aimed as an update from the first one– was run on PubMed and Embase.com from inception to October 4^th^, 2023. Search terms varied slightly between searches and included controlled terms (MeSH-terms) as well as free text terms. The following terms were used (including synonyms and closely related words) as index terms or free-text words: ‘oral tissues’ and ‘sex hormones’. On the first search, a filter was used to exclude animal studies. On the second search, additional filters were used to further limit the results. The search was performed without date or language restrictions. The full search strategy can be found on **Appendix 1**.

### 2.2 Screening process and eligibility criteria

References resulting from the search strategy were imported in Rayyan (26), an online application used for screening articles simultaneously and independently by the reviewers. Screening of the search results was done independently by three reviewers (P.C., R.N. and J.B). Articles were initially screened based on title and later based on abstract. On each step, discrepancies were discussed until reaching an agreement. The same process was followed for full-text review. References were managed using Endnote 20 (27). Studies were ultimately verified for their eligibility for inclusion based on full text.

Studies that met the following criteria were included in this scoping review: (1) *in vitro* or *in vivo* studies; (2) information about the physiological role of sex steroid hormones progesterone, estrogen and testosterone and their precursors in periodontium, dental structure, mucosa, salivary glands, local immune response and orofacial perception. Studies reporting on: (1) puberty; (2) menstrual cycle; (3) pregnancy; (4) menopause; (5) andropause; (6) pathological hormonal imbalances; (7) hormonal therapy; (8) other non-hormonal therapy; (9) cancer; (10) microbiome; (11) biofilm; (12) animal studies; (13) discussion papers; (14) letters to the editor; (15) reviews that did not specify the search methodology and; (16) case-control studies, were excluded.

### 2.3 Data extraction and summary

In this review, the evidence was divided into four distinct categories: (1) Periodontium; (2) Dental structure; (3) Mucosa; and (4) Salivary glands. Their interaction with progestagens, androgens and estrogens were summarized.

## 3 Results

### 3.1 Selection of evidence

**Figure 2:**
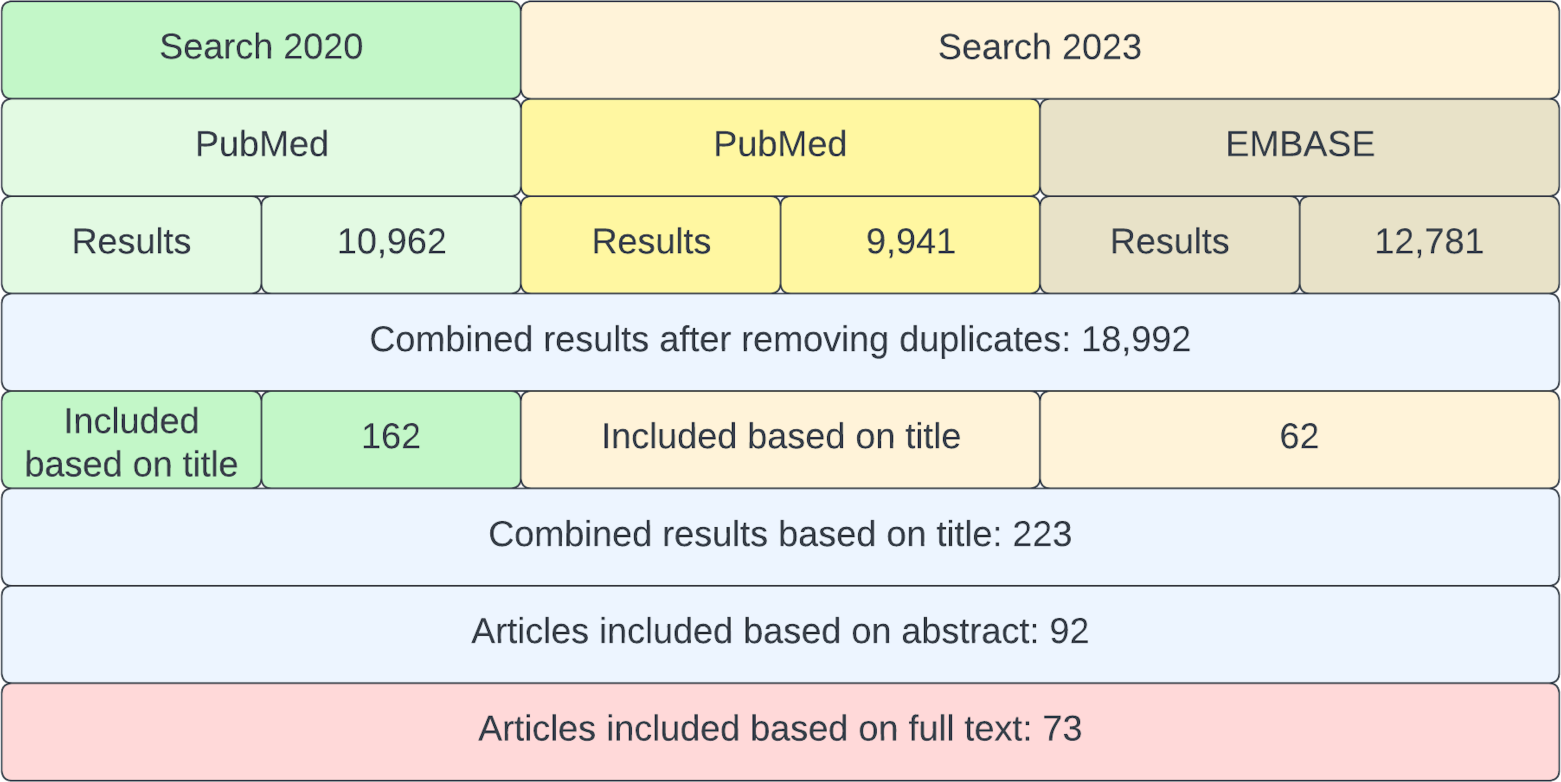
Flowchart of the screening process. In green, the search from 2020 and in yellow/brown, the search from 2023. Finally, all articles from both searches were combined and assessed for inclusion.

The screening process and the number of selected articles are summarized in **Figure 2**. The first search yielded 10,962 records in PubMed. The second search yielded 22,722 results in total (9,941 in PubMed and 12,781 in EMBASE). After comparing both searches from 2020 and 2023 and removing the duplicates using the computer software DedupEndNote (28), our combined searches yielded 18,992 results. From these results, 162 articles were selected by title (from the first search) and 62 (from the second search). During the next screening phase, 92 articles were selected by abstract. Out of 92 records, 19 records were excluded for the following reasons: (1) no full text available; (2) no direct relation to oral health and; (3) main focus did not match the objectives of this scoping review; (4) not in English, Dutch, Spanish, Italian or Portuguese language. The remaining 73 records were considered included in this review.

## 4 The oral cavity as a target organ

Periodontal tissues and their response to changes in SSH –such as during pregnancy– have for long been the main focus of oral endocrinology research (29). This special interest on periodontal tissues can be attributed to the clinical changes and associated detrimental consequences for oral and general health (30). The understanding of the physiology and physiopathology of these phenomena becomes fundamental in disease treatment and prevention. However, and as it has already been exposed above, SSH are extremely versatile molecules with a great variety of target organs, which is also reflected in the oral cavity. In addition to the effects on periodontal tissues during periods of fluctuating hormonal levels, SSH have been reported to interact with practically every other tissue type present in the oral cavity. There is evidence of direct effects of SSH on teeth, periodontium, oral mucosa and salivary glands. Also, SSH’s role has been described in amelogenesis, odontogenesis, bone metabolism and local immune response.

In this review, the evidence is divided into four categories: (1) Periodontium; (2) Dental structure; (3) Mucosa; and (4) Salivary glands. Their interaction with progestagens, androgens and estrogens will be summarized. Human and *in vitro* studies were included where only physiologic effects were demonstrated. Studies addressing pathological effects or treatment are beyond the scope of this review.

### 4.1 Periodontium

The periodontium includes the tissues supporting and surrounding the teeth. These tissues are the gingiva, periodontal ligament (PDL), cementum and the alveolar bone.

The gingiva is mainly composed of both epithelial cells and fibroblasts (31). PDL is a connective tissue structure surrounding the root of the tooth, connecting it to the alveolar bone. Cells present in the PDL include periodontal ligament stem cells (PDLSCs), fibroblasts, endothelial cells, cementoblasts, osteoblasts, osteoclasts, tissue macrophages, and stratified epithelial cells. PDLSc have differentiation potential, which has proved to be essential in periodontal tissue repair and regeneration (32). Cementum is the calcified mesenchymal tissue surrounding the root of the tooth which is secreted by cementoblasts. This tissue allows the insertion of the PDL fibers to the tooth and the alveolar bone (33) which is the osseous tissue of the maxilla and the mandible, forming the tooth socket that surrounds the tooth (34). The existing studies reporting interactions between SSH and the periodontium are listed in **Table 1**.

**Table 1:** Studies reporting effects and interactions of SSH and periodontium.

	Progestagens	Androgens	Estrogens
*Gingiva*			
*Receptors*	PR in hGF (35).	DHT receptors in the cytoplasm of gingival tissue (22) and AR in gingival tissue (36) and gingival fibroblasts (24, 37).	Expression of ER (23) and ERβ on gingival epithelial cells (38, 39).
*Metabolism*	Metabolism of progesterone by 5α-reductase, 3β-hydroxysteroid-dehydrogenase, and 20α-hydroxysteroid-dehydrogenase (40). Metabolism of progesterone with varied by-products as a result (41). Progesterone modulates androgen metabolism in hGF (42, 43).	Conversion of androstenedione to testosterone by 17β-hydroxy-C_19_-steroid oxidoreductase (44). Metabolism of testosterone and androstenedione into reduced forms in hGF (42, 43) and in male and female gingival tissue (45). Identification of different testosterone-reducing enzymes in gingiva (46).	Conversion of estrone to estradiol by 17β-hydroxy-C_18_-steroid oxidoreductase (47). Estradiol modulates androgen metabolism in hGF (42, 43).
*Cellular growth, differentiation, proliferation and migration*		Testosterone stimulates proliferation of hGF (36).	Estrogen induces proliferation of hGF (48). Estrogen levels correlate to the expression of cytokeratin 5 (49).
*Cytokine production and inflammation markers*	Progesterone inhibits (Gornstein et al., 1999; Lapp et al., 1995) or enhances (Yokoyama et al., 2005) IL-6 production in hGF. Progesterone enhances IL-8 and VEGF production (50). Progesterone upregulates the expression of COX-2 in hGF (51).	IL-6 expression in oral fibroblasts is inhibited by testosterone (36, 37, 52) and DHT (37, 52). Testosterone downregulates the expression of IL-17 after 24 hours of exposure (53). Testosterone inhibits prostaglandin formation (54).	Production of vascular endothelial growth factor (VEGF), interleukin IL-6 and IL-8 by hGF increased following stimulation with estradiol (50). The pro-inflammatory effects on hPDL cells induced by LPS (secretion of TNF-alpha, IL-1beta, IL-6, and receptor activator of NF- B ligand (RANKL)) are reversed by Estradiol. Also, estradiol upregulates osteoprotegerin expression, thus attenuating the osteoprotegerin vs. RANKL ratio (55). 17β-estradiol upregulates the expression of COX-2 in hGF (51).
*PDL*			
*Receptors*	Ability of hPDLCs to bind progestins, suggesting the presence of PR (56) and expression of PR (57)	AR in periodontal tissue and hPDL fibroblasts (24, 37, 53).	Expression of estradiol (56) and estrogen receptors (53, 58), expression of ERβ in the nuclei (59) and mitochondria of hPDLCs (60) and expression of only ERα (61), ERβ (62) or both ERα and ERβ in hPDLCs (63, 64). hPDLSCs express ERα and ERβ (65, 66). Upregulation of ERβ during osteogenesis (63).
*Metabolism*	Metabolism of testosterone and androstenedione (67).		Collagen and DNA synthesis of hPDLCs is not influenced by estradiol (68)
*Cellular growth, differentiation, proliferation and migration*	Progesterone stimulates hPDLCs proliferation and differentiation under osteogenic conditions. Also, progesterone can enhance alkaline phosphatase activity and the expression of genes coding for mineralization processes (57).		Estrogen downregulates osteoclast formation (69), enhances osteocalcin production (70, 71), alkaline phosphatase activity (70) and mineralized nodule formation of hPDLCs (72). Estrogen also induces osteogenic differentiation of hPDLSCs via ERα (65) and ERβ (66). Estradiol inhibits the growth rate of hPDLCs in a dose dependent manner (56), increases their proliferation (62, 73) and enhances osteogenic differentiation (62, 64, 73). Estradiol also increases expression of osteoprotegerin (OPG) and decreases expression of nuclear factor-kappa β ligand (RANKL) in hPDLCs via ERβ (61). PDLSCs, when supplemented with estradiol, show odonto/osteoblast differentiation capacities (74)
*Cementum*			
			Expression of ERα and ERβ (75). 17- β Estradiol significantly increases proliferation of hCDCs (76).
*Alveolar bone*			
	See *Cellular growth, differentiation, proliferation and migration* of PDL for overlap between hPDLCs and alveolar bone on: differentiation, alkaline phosphatase activity and expression of genes related to the mineralization process.		See *Cellular growth, differentiation, proliferation and migration* of PDL for overlap between hPDLCs and alveolar bone on: osteogenic differentiation, osteoclast formation. Osteocalcin production, alkaline phosphatase activity, mineralized nodule formation, upregulation of OPG and down-regulation of RANKL.

AR, androgen receptor; DHT, 5α-Dihydrotestosterone; ER, estrogen receptor; hGF, human gingival fibroblasts; hPDL, human periodontal ligament; hPDLCs, human periodontal ligament cells; hPDLSCs, human periodontal ligament stem cells; hCDCs, human cementum-derived cells; OPG, osteoprotegerin; PDL, periodontal ligament; PR, progesterone receptor; RANKL, nuclear factor-kappa β ligand; VEGF, vascular endothelial growth factor.

#### 4.1.1 Receptors

In order for the periodontium to communicate with molecular messengers such as SSH, specific receptors should be present in the target tissues. Several studies have demonstrated the presence of SSH receptors in gingiva, PDL, cementum and alveolar bone. In gingival tissue, receptors for progesterone (35), androgens (22, 24, 36, 37) and estrogens (23, 38, 39) have been described in both epithelial cells and fibroblasts.

PDL expresses receptors for progesterone (57), androgens (24, 37, 53) and estrogens (53, 56, 58–66).

Cementoblasts are known to express estrogen receptors α (ERα) and β (ERβ) (75).

Osteoblast-like cells –responsible for synthetizing bone tissue– express progesterone (77), estrogen (78) and androgen receptors (79). This also applies to alveolar bone. Previous publications have extensively reviewed the expression of sex steroid receptors and the physiology of the interaction between bone tissue and SSH (80, 81). Addressing this topic falls outside the scope of this review. Therefore, only papers specifically focusing on alveolar bone will be discussed.

All four tissues of the periodontium express receptors for SSH, confirming their susceptibility to sex steroid hormones.

#### 4.1.2 Metabolism

Periodontal cells present enzymes capable of metabolizing SSH, resulting in the conversion of sex hormones into different by-products. The first *in vitro* evidence of the presence of enzymes in gingiva able to metabolize progesterone was reported in 1971 (82). El Attar, demonstrated the presence of 5α-reductase, 3β-hydroxysteroid-dehydrogenase, and 20α-hydroxysteroid-dehydrogenase in gingival tissue from patients with periodontitis, concluding that the metabolism and degradation of progesterone could contribute to the state of health of the gingiva. The capacity of healthy gingiva to metabolize progesterone (40, 41), androgens (44–46) and estrogens (47) has also been reported. Interestingly, inflamed gingiva has proved to be significantly more active than healthy gingiva at metabolizing SSH.

Notably, the rate of metabolic conversion of certain SSH can in turn be influenced by the presence of other SSH (42). In cultured inflamed human gingival fibroblasts (hGF) from primary cells, supplementation of different concentrations of either estradiol or progesterone have shown antagonistic effects. While estradiol stimulated androgen conversion, progesterone inhibited it. When combined, an initial increase in androgen conversion was followed by an inhibitory effect, directly related to the increasing concentration of both estradiol and progesterone combined (43).

Other compounds such as cytokines, growth factors, prostaglandins and medication are capable of influencing the synthesis of SSH by gingival cells by either promoting (83–87) or inhibiting (87) the synthesis of SSH by gingival cells.

PDL cells are known to metabolize testosterone and androstenedione *in vitro*, indicating the presence of reductase enzymes (67).

Bone metabolism and SSH have been previously reviewed (88) and will not be addressed. Shortly, SSH metabolism by osteoblast-like cells has been reported (89, 90) and there is enough evidence that confirms a central role of SSH and bone resorption and apposition (91).

Based on the existing evidence, gingival cells and PDL are capable of metabolizing SSH into different by-products. Also, gingival cells are susceptible to both the presence of SSH and diverse external factors, which can modulate SSH metabolism. Research on PDL and hormone metabolism is still very limited, but the existing evidence is in line with what has been reported for gingival tissue. There is to our knowledge no available evidence on metabolic activity of SSH by cementoblasts.

#### 4.1.3 Cellular growth, differentiation, proliferation and migration

Evidence about the expression of SSH receptors and metabolic enzymes in the periodontium supported the hypothesis that SSH were capable of inducing physiological changes in periodontal tissues. This was eventually tested in the early 2000s by different research groups that assessed the response of human gingival fibroblasts (hGF) to different SSH during different cellular processes (36, 48). Effects such as changes in cellular growth, differentiation, proliferation and migration were found for all three types of sex steroids. In gingival tissue, direct effects of testosterone and estrogen have been described. Testosterone can stimulate cellular proliferation and migration (36) and estrogen has been described to induce hGF proliferation and decrease protein production (48).

PDL cells have been the focus of broader research. As previously mentioned, periodontal cells have differentiation potential and have thus the ability to form bone, cementum and collagen fibers (32). PDL cells bridge the interaction between tooth and alveolar bone and by this, they play an active role in bone-remodeling and the physiology of the periodontium. SSH progesterone and estrogen can modulate this process. When exposed to progesterone *in vitro*, PDL cells have shown enhanced proliferation and osteogenic differentiation (70). Different responses of PDL cells to estradiol have been described. Estradiol has been found to either inhibit the growth of PDL cells in a dose-dependent manner (56) or not exerting any measurable effect on proliferation and collagen formation (68). Contrary to these results, estrogen, like progesterone, has been reported to enhance the proliferation and osteogenic differentiation of PDL cells (62, 73) and also mediate osteogenic differentiation in PDL stem cells (65, 66). Also, estrogen is capable of inhibiting the formation of osteoclast-like cells, which are responsible for bone resorption in cocultures of PDL fibroblasts and peripheral blood mononuclear cells (69). In cementoblasts, estradiol has been reported to enhance their proliferation (76).

Additional effects of SSH on PDL cells have also been described. Estrogen has been reported to promote osteocalcin production (70, 71) and mineralized nodule formation (72). Additionally, estrogen has been described to enhance alkaline phosphatase activity (61) and increase the expression of osteoprotegerin while decreasing the expression of RANKL in PDL cells via ERβ (61). These findings indicate an active role of estrogen in bone-remodeling and the physiology of the periodontium.

The described effects support the hypothesis of a relevant physiological role in homeostasis of SSH in the oral cavity.

#### 4.1.4 Sex steroid hormones and cytokine production in the periodontium – a bidirectional interaction

Several studies have reported immunomodulating effects of SSH via different mechanisms. Also, different oral cells are capable of producing different cytokines when exposed to sex steroids. A number of studies have reported the up and downregulation of cytokine production by SSH in gingival fibroblasts with contradictory results. Progesterone has been described to inhibit the production of IL-6 in a dose-dependent manner (37, 92) but also –together with estradiol– increase the production of IL-6 and IL-8 together with an increased secretion of vascular endothelial growth factor (VEGF) (50). This has also been reported in non-oral cell types (93, 94). Also, in immune challenged gingival fibroblasts, progestin and estradiol are capable of downregulating various inflammatory cytokines (95).

Androgens are also capable of modulating the production of certain cytokines and prostaglandins by gingival fibroblasts. Testosterone (36, 37) and DHT (37, 52) downregulate the production of IL-6. Testosterone has also been described to downregulate the synthesis of prostaglandins (54), suggesting an anti-inflammatory effect in gingival tissue. In PDL cells, testosterone and estradiol seem capable of downregulating the production of IL-17 (53).

Cytokine expression and its modulation by SSH in LPS-stimulated PDL cells has also been studied. Shu et al. tested the effects of estradiol on both spontaneous and LPS-stimulated cytokine expression (55). Results showed that estradiol did not have a big effect on spontaneous cytokine production, but it did actively regulate cytokine expression when co-cultured with LPS. TNF-α, IL-1β, IL-6 and RANKL which are normally upregulated by LPS, were significantly suppressed in the presence of estradiol. Osteoprotegerin (OPG) was upregulated. This suggests a modulation of the stimulatory effects of LPS on these cytokines. This phenomenon has also been observed in the expression of certain chemokines, which are either up or downregulated by estradiol when PDL cells are challenged by exposure to LPS (96).

Just like SSH are capable of regulating cytokine production, certain cytokines have been described to modulate the conversion rate of SSH in periodontal inflamed tissue. In particular, IL-1 has been described to enhance the conversion of androstenedione and testosterone to dihydrotestosterone (DHT) –a potent androgen– in gingival tissue and PDL cells (83, 97). Although research regarding this phenomenon in healthy tissues is not available, it is important to acknowledge the possibility that healthy tissues may respond in a similar manner.

An increase in androgen conversion (DHT and 4-androstenedione) by healthy gingival tissue in the presence of prostaglandins was reported in one study (86). Prostaglandins are lipid-derived molecules involved in the regulation of inflammation. Conversely, another study reported that exposure of gingival fibroblast to either progesterone, estradiol or both, upregulated the expression of COX-2, an enzyme involved in the synthesis of prostaglandins (51). These responses by gingival cells provide further insight in the effects of SSH and adaptative changes in hormone metabolism during inflammation.

### 4.2 Dental structure

The tooth is composed of four distinct tissues, enamel –the outer layer of the tooth– and the underlying layers dentin, pulp and cementum.

Each of these tissues is composed of distinct cell types. Enamel is the mineralized tissue secreted by ameloblasts. Dentine is a matrix secreted by odontoblasts. Pulp is composed by a complex multicellular organization including fibroblasts as well as odontoblasts, immune cells, neural fibers, amongst others. Cementum is secreted by cementoblasts (33). In all these tissues, direct or indirect evidence has been found on the presence of SSH receptors for one or more sex steroids. The existing evidence is listed in **Table 2**.

**Table 2:** Studies reporting effects and interactions of SSH on the dental structure.

	Progestagens	Androgens	Estrogens
*Enamel*			
*Receptor polymorphisms and effects on the enamel*			Genetic polymorphisms in ER are associated with developmental defects of the enamel (98), higher caries experience (99) and a higher incidence of fluorosis in children (100, 101).
*Pulp*			
*Receptors*	Pulp fibroblasts and odontoblasts expressed PR (102)	Pulp cells expressed AR (103). Freshly isolated pulp tissue expressed AR, which was significantly more abundant in male subjects. Its expression increased when exposed to estradiol or androstenedione, while exposure to testosterone decreased its expression (104).	hDPC expressed ERα, ERβ1 and ERβ2. During osteogenic differentiation of hDPCs, ERβ1 and ERβ2 were upregulated and ERα was downregulated (105). Pulp cells expressed ERα and ERβ (103). Pulp cell cultures expressed ERβ (104). Odontoblasts, endothelial cells and Schwan cells (all derived from pulp tissue) expressed ERα (106). Odontoblasts and endothelial pulp cells expresser ER (107).
*Cellular differentiation*		DHT upregulated the expression of several genes involved in odontogenesis and odontoblast differentiation in hDPCs (103).	Estradiol enhanced hDPSCs’ ALP activity, mineralization capacity and promoted odonto/osteogenic differentiation (108). It also increased proliferation of hDPCs and upregulated odontoblastic differentiation markers (109). When inducing the overexpression of ERα in hSCAPs ALP activity, mineralization capacity and odonto/osteogenic differentiation were significantly increased (110). hDPSCs, hSCAP and hDFSCs show odonto/osteogenic differentiation potential when supplemented with estradiol (74). Estradiol up-regulated ALP activity, mineralization capacity and odonto/osteogenic markers in hSCAPs (111). Estradiol induced up-regulation of several genes involved in odontogenesis and odontoblast differentiation in hDPCs (103). Estradiol enhanced expression of osteoprotegerin (OPG) in hPDLCs via a membrane-bound receptor (112).

ALP, alkaline phosphatase; AR, androgen receptor; DHT, 5α-Dihydrotestosterone; ER, estrogen receptor; hDPCs, human dental pulp cells; hDPSCs, human dental pulp stem cells; hSCAPs, stem cells from the apical papilla; hDFSCs, human dental follicle stem cells; OPG, osteoprotegerin; PR, progesterone receptor.

#### 4.2.1 Receptors

Evidence of SSH receptors on enamel development and maturation is scarce. The presence of androgen (113) and estrogen receptors (114) in ameloblasts and their involvement in the developmental process of enamel has been reported in rats. In humans, only indirect evidence has been described, linking the presence of estrogen receptor’s polymorphisms to the incidence of clinical changes in the enamel. Developmental defects of the enamel (DDE) (98), a higher caries incidence (99), a higher incidence of fluorosis in general (100) and in high-fluoride-exposure areas (101) indicate an important role of estrogen and estrogen receptor in amelogenesis.

In pulp tissue, progesterone (102), androgen (103, 104) and estrogen receptors (103–107) have been identified.

The expression of androgen receptors in the pulp can apparently change when exposed to certain SSH. *In vitro*, the addition of androstenedione or estradiol increased the expression of androgen receptors (AR). On the contrary, the addition of testosterone reduced its expression (104). This indicates an active role of SSH in the pulp responsiveness to androgens, not only by direct interaction with sex steroid receptors but also by manipulating the expression of AR. Pulp estrogen receptors can also be up or downregulated during osteogenic differentiation (105).

#### 4.2.2 Cellular growth, differentiation, proliferation and migration

As previously mentioned, pulp tissue includes different cell types including stem cells. It has been reported that the differentiation potential of these pulp stem cells can be enhanced by estradiol. An increased alkaline phosphatase activity (ALP), mineralization capacity and upregulation of odonto/osteogenic differentiation markers have been described (74, 108). This has also been observed in stem cells from the apical papilla (portion at the apex of the root) of pulp tissue from immature teeth (74, 110, 111) and to a lower degree in stem cells from the dental follicle (tissue surrounding an unerupted tooth) (74). In human dental pulp cells, estradiol has been also reported to increase proliferation (109) as well as odontoblastic differentiation (103, 109). Genes directly involved in odontogenesis and osteoblast differentiation such as *AMBN, IFT88, TP63* are upregulated by estradiol and the androgen DHT (103). An increase of osteoprotegerin (OPT) –an essential protein in bone remodeling and homeostasis– has also been reported to be susceptible to estradiol, which enhances its expression (112).

Most studies addressing the effects of SSH on pulp tissue have focused on the effects of estradiol, possibly motivated by the onset of bone resorption as estrogen decreases (115). Estradiol enhances odonto/osteogenic differentiation in different cell types of the pulp and surrounding tissues, evidencing its role in tissue formation, repair and adaptation to changing external factors, all important aspects in homeostasis.

### 4.3 Oral mucosa

The oral mucosa is an important protection barrier against microbes, toxins and mechanical and chemical damage through its physical and immunological functions (116). Like gingival tissue, oral mucosa stems from two distinct embryonic layers. The epithelial layer originates from the ectoderm and the deeper layers stem from the neural crest ectomesenchyme (endoderm) (117). The interaction of these layers is fundamental for the proper development and function of the oral mucosa, and sex steroid hormones also play a role in these processes. The existing evidence is listed in **Table 3**.

**Table 3:** Studies reporting effects and interactions of SSH and oral mucosa.

	Androgens	Estrogens
*Mucosa*		
*Receptors*	Buccal mucosal cells express AR (118).	ER is expressed in buccal mucosa of pre- and post-menopausal women (119). ERβ is expressed in buccal mucosa of men and women (39).
*Wound healing*	Wound healing *in vivo* decreases with age regardless of sex and is significantly slower in females than in males regardless of age (120). Wound healing *in vivo* is faster in young individuals with low testosterone levels. In post-menopausal women, a faster healing correlates with higher testosterone levels in blood (121).	
*Cytokine production*		Estradiol downregulates expression of hBD-2, IL-6 and IL-8 in hOMEC (122).

AR, androgen receptor; ER, estrogen receptor; hOMEC, human oral mucosal epithelial cells.

#### 4.3.1 Receptors

Traditionally, oral mucosa has not been regarded as a target tissue for SSH in healthy individuals. The existing studies have focused on complaints manifesting around and after the onset of menopause, where women experience discomfort in different oral mucosal tissues, and not on the physiological role of SSH in oral mucosa (123). Limited research has been done on the expression of SSH receptors in oral mucosa in humans with some studies reporting on the presence of androgen and estrogen receptors. Androgen receptors have been described in buccal mucosa of healthy individuals from both sexes (118). Estrogen receptors have been described in buccal mucosa of young and post-menopausal women (119) and in buccal mucosa of men and women (39). The limited available evidence supports the notion that oral mucosa is also a target tissue for SSH.

#### 4.3.2 Wound repair

The process of wound repair has been previously studied, and histatins have been found to play a fundamental role in restoring the integrity of damaged mucosa (124). Nonetheless, some studies have addressed the role of SSH and mucosal wound healing. This has been tested *in vivo* by inflicting a small wound on the hard palate of 212 individuals from both sexes ranging from 18–35 years old and 55–88 years old (120). As expected, age negatively influenced wound healing regardless of sex. However, wound healing in females was significantly slower regardless of age. A follow-up to this study used the same methodology in a larger cohort (121). A group of 329 individuals (age range 18–43) and a smaller group of 93 individuals (age range 55–88) were inflicted a small wound on the hard palate and videographed every 24 hours for 1 week. Hormone levels in blood were also measured. Results showed that lower testosterone levels in the younger group related to faster wound healing whereas higher testosterone levels in post-menopausal women related to faster healing times.

In cutaneous wound healing, women have a significant advantage compared to males (125). In other mucosal surfaces, estrogen has been put forward as a protective factor in mucosal injury (126). This data suggests that sex hormones and wound repair could be tissue specific. When contrasting the existing *in vivo* research with studies assessing wound healing in other tissues it is reasonable to suggest that wound healing is a complicated process that not only involves migration of cells but also the host’s immune response, which –as it will be soon discussed– can be modulated by SSH.

#### 4.3.3 Sex steroid hormones and cytokine production in oral mucosa

As previously mentioned, the oral mucosa has a gatekeeper function which is extremely important for the host. Moreover, the oral mucosa also exerts a regulatory control over the local immune response (127). This modulation of the local immune response has been reported during the interaction of the commensal oral flora with the mucosal surfaces (128), during recurrent mechanical damage (129) and when exposed to mucosal sensitizers (130), to name some. However, little is known about the potential role of hormones during this process.

What we know so far is that estradiol might have a regulatory effect on the expression of certain cytokines in oral epithelial cells (122). When these cells were exposed to IL-1β, the mRNA and protein expression of Human β-Defensin 2 (hBD-2)– an antimicrobial peptide–, IL-6 and IL-8 were upregulated. In the presence of estradiol, a downregulation of these molecules was observed, suggesting and anti-inflammatory effect of estradiol in oral mucosal epithelial cells.

### 4.4 Salivary glands

In humans, saliva is produced by major and minor salivary glands, which originate from the oral epithelium. Major salivary glands consist of three pairs of glands, namely the parotid, submandibular and sublingual glands. Minor salivary glands can be found throughout the oral cavity (131). The effect of sex steroids on the structure, salivary production amongst others have been widely studied using animal models (132–135). In humans, some studies have been published on the effects of different SSH on salivary glands. These are summarized in **Table 4**.

**Table 4:** Studies reporting effects and interactions of SSH and salivary glands.

	Progestagens	Androgens	Estrogens
*Salivary glands*			
*Receptors*		AR are expressed in parotid, submandibular and minor salivary glands (136). AR are expressed in minor sebaceous salivary glands (137).	ER is expressed in submandibular, parotid and minor salivary glands of pre- and post-menopausal women (119). ERβ is expressed in submandibular, parotid and minor salivary glands of men and women in acinar and ductal cells (39). Expression of ERα, ERβ1 and ERβ2 was detected in minor salivary glands (138).
*Metabolism*	Parotid and submandibular glands are capable of metabolizing progesterone and testosterone *in vitro* (139).		Cultured salivary gland cells exposed to estrogen showed a significant inhibition of IFN-γ-induced ICAM-1 expression (138).

AR, androgen receptor; ER, estrogen receptor.

#### 4.4.1 Receptors

Androgen (136, 137) and estrogen receptors (39, 119, 138) have been reported in major and minor salivary glands.

The existing literature confirms the susceptibility of salivary glands to androgens and estrogens.

#### 4.4.2 Metabolism

Only one study has addressed the capacity of salivary glands to metabolize SSH. Blom et al. measured several sex-steroid by-products after incubating salivary gland samples with progesterone and testosterone *in vitro* (139). Based on the detected metabolites, it was concluded that these cells presented steroid-reductase, dehydrogenase and hydroxylase enzymes.

#### 4.4.3 Sex steroid hormones and cytokine production in salivary glands

Cytokine production and modulation by SSH is salivary glands is an extremely understudied topic. To our knowledge, only inhibition of IFN-γ-induced ICAM-1 expression by estrogen was reported in one study (138).

## 5 SSH-mediated tissue interactions and maintenance of oral health

**Figure 3:**
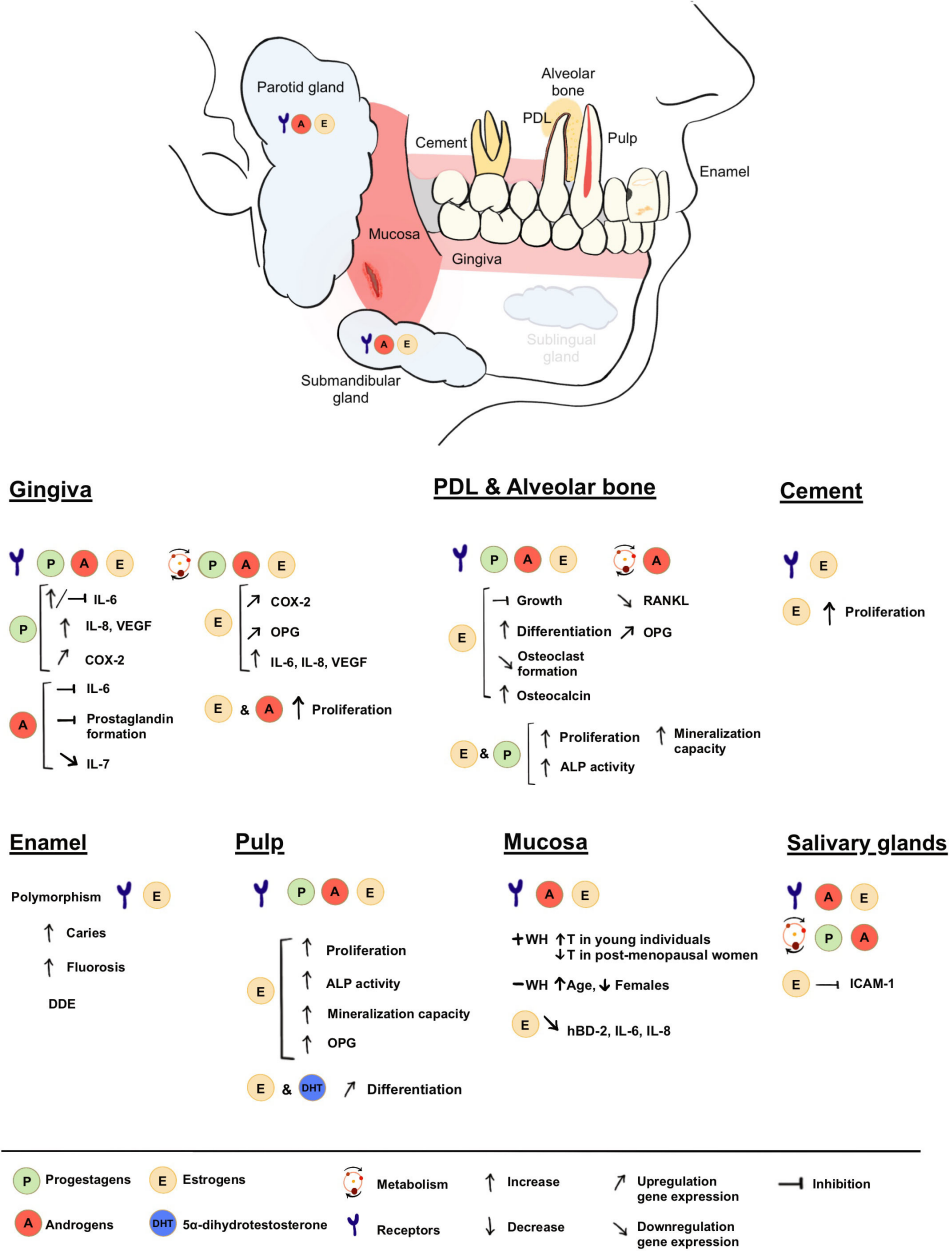
Summary of presence and roles of SSH in the oral cavity.

In this review we have provided a comprehensive overview of the scientific literature concerning the interactions between sex steroid hormones and oral tissues. By this, we aimed to clarify the physiological roles of sex steroids within the oral cavity, their role in oral homeostasis and in the development and function of different oral tissues. Our analysis of the existing body of evidence confirms the enormous versatility of SSH in contributing to various aspects of oral health maintenance (**Figure 3**). Their contribution can be summarized as follows: (1) Sex steroids exert a significant influence on periodontal health by playing an active role in bone remodeling and its immune response; (2) Several sex-hormone-mediated inflammatory mediators are up or downregulated by SSH, providing protection to external disturbances; (3) Sex steroids significantly contribute to the proper development of enamel, a fundamental process that ensures adequate protection of the dental structure; and (4) SSH assume a vital role in preserving mucosal health and integrity by promoting vascularization and regulation of inflammation, thus facilitating optimal tissue turnover and wound healing. These processes and balance could not be attained without the finely orchestrated interplay between SSH and their biosynthesis, conversion, transport, and consequent signaling to achieve the desired biological effects.

All things considered, it is evident that sex steroid hormones assume a pivotal role in the physiological maturation and maintenance of oral tissues in the absence of physiological disbalances.

But what happens when the balance is disrupted?

In the event of abrupt hormonal fluctuations, the systemic repercussions are notable. Temporary hormonal perturbations can be assimilated by the body: its inherent resilience and immune fitness allow a swift return to homeostasis without significant consequences (e.g. pregnancy). However, the persistence of such hormonal alterations can hinder the body’s adaptive capacity, progressively weakening physiological functions and eventually resulting in further instability of the system and the onset of disease. Moreover, disease does not affect males and females in the same way.

### 5.1 Sexual dimorphism in oral disease

Numerous diseases exhibit variations in prevalence, progression, and manifestation between males and females. These and other biological differences between males and females are referred to as sexual dimorphism. Regarding oral diseases, males are more prone to severe periodontal disease than females (140, 141). This may be attributed –in part– to differences in hormonal levels and their impact on immune modulation. Several examples for different pathologies can be found in the literature.

A recent systematic review investigated the expression of sex steroid hormones (SSH) in oral squamous cell carcinoma (OSCC), a condition observed to be twice as prevalent in males than females (142). This review concluded that sex hormones and sex steroid receptors play a significant role in influencing the physiopathology of OSCC.

Other pathologies such as Sjögren syndrome (SS), burning mouth syndrome (BMS) and temporomandibular disorders (TMD) are more prevalent in females than in males (143–145). SS is an autoimmune condition that affects the lacrimal and salivary glands, negatively affecting saliva production, which results in severe dry-mouth symptoms (146). Low levels of dehydroepiandrosterone (DHEA) have been associated with SS manifestation and hormonal supplementation has proved to improve dry mouth symptoms (147). BMS has also been associated with lower levels of DHEA (148) and TMD has been associated with low estrogen levels (149) and estrogen receptor gene polymorphism (150).

The role of SSH on the incidence and progression of conditions in the oral cavity is not completely understood, but sexual dimorphism in disease has been observed in several conditions (151).

Aside from genetical and behavioral aspects that also influence the differential response to disease (151), factors such as SSH receptors, the interplay and balance between different sex steroids and their conversion in biological tissues play an active role on disease (152, 153). In endometrial cancer, the loss of progesterone and estrogen receptors is associated with an advanced disease stage (154) and hormonal imbalances can result in changes in gene expression, further influencing disease progression (155). Also, changes in SSH signaling can contribute to lung disease (156) and changes in the enzymatic expression of aromatase in tumor-associated macrophages have been found to correlate with the severity of breast cancer (157). This also exemplifies the influence and relevance steroidogenesis, and the subsequent cascade of events can have in the maintenance or loss of homeostasis.

Changing hormone levels are closely related to aging (158). Aging increases the risk and severity of various diseases, and the oral cavity is no exception (159). This is particularly evident as SSH levels sharply decrease with the onset of menopause in women and gradually decline due to reduced androgen levels in men (160, 161). These decrease in SSH significantly impact the immune system, among other effects (162). Several studies have been conducted on the changes of SSH, sex, aging and oral diseases (163–167), confirming the significant influence of these factors on oral homeostasis.

Sexual dimorphism is present in several diseases and therefore sex differences should be included as a variable in research. Recognizing and understanding the different roles of SSH in oral physiology are fundamental for preventing and treating oral diseases.

### 5.2 Clinical implications and future challenges

This review has focused on the role of SSH in the maintenance of oral health and their involvement in different physiological processes in the oral cavity. Nevertheless, the microbiome and its influence on the maintenance of homeostasis in the oral cavity has not been mentioned. It is beyond the scope of this review to discuss this; however, it is important to stress that both homeostasis and dysbiosis are multifactorial biological processes. Therefore, future research should also consider the interplay between SSH, the oral microbiome and the host, and how they influence homeostasis and dysbiosis in the oral cavity in humans.

It is important to emphasize that hundreds of studies on this topic have been conducted using animal models. These studies have significantly contributed to our understanding of how sex steroid hormones interact with complex biological systems. However, results from animal studies are not always easily translatable to human physiology. We therefore focused this scoping review only on studies related to humans. Nonetheless, animal studies have served as a crucial steppingstone, allowing several hypotheses to be tested in humans, as presented in this paper.

## 6 Conclusion

Sex steroid hormones are an overlooked yet fundamental factor in oral homeostasis. They play important roles in the development and function of the periodontium, mucosa, dental structure and salivary glands. Nevertheless, it is important to acknowledge that the onset, progression and ultimate resolution of disease are multifactorial processes, and the presence of hormonal imbalances alone is insufficient to account for their etiology and eventual reinstatement of homeostasis. However, it is important to consider sex steroid hormones as a relevant factor in oral health maintenance, which is complex and can vary from person to person. Hormone levels can fluctuate due to factors like age, hormonal treatments and medical conditions. Research on this topic is ongoing, and the precise mechanisms and clinical implications are areas of active study in the field of dentistry and oral health. Dentists and healthcare providers should consider these hormonal factors when assessing and treating oral health conditions.

## Author contributions

PC: Conceptualization, Data curation, Formal analysis, Investigation, Methodology, Visualization, Writing – original draft. BK: Supervision, Writing – review & editing. LS: Methodology, Writing – review & editing. MV: Supervision, Writing – review & editing.

## References

[1] Payne AH Hales DB. Overview of steroidogenic enzymes in the pathway from cholesterol to active steroid hormones. Endocr Rev. (2004) 25:947–70. doi: 10.1210/er.2003-0030 15583024

[2] Asarian L Geary N. Modulation of appetite by gonadal steroid hormones. Philos Trans R Soc Lond B Biol Sci. (2006) 361:1251–63. doi: 10.1098/rstb.2006.1860 PMC1642706 16815802

[3] Pöllänen E Kangas R Horttanainen M Niskala P Kaprio J Butler-Browne G. Intramuscular sex steroid hormones are associated with skeletal muscle strength and power in women with different hormonal status. Aging Cell. (2015) 14:236–48. doi: 10.1111/acel.2015.14.issue-2 PMC4364836 25645687

[4] Gautier A Bonnet F Dubois S Massart C Grosheny C Bachelot A. Associations between visceral adipose tissue, inflammation and sex steroid concentrations in men. Clin Endocrinol. (2013) 78:373–8. doi: 10.1111/j.1365-2265.2012.04401.x 22469460

[5] Wawrzkiewicz-Jałowiecka A Lalik A Soveral G. Recent update on the molecular mechanisms of gonadal steroids action in adipose tissue. Int J Mol Sci. (2021) 22:5226. doi: 10.3390/ijms22105226 34069293 PMC8157194

[6] Krum SA. Direct transcriptional targets of sex steroid hormones in bone. J Cell Biochem. (2011) 112:401–8. doi: 10.1002/jcb.22970 PMC3070194 21268060

[7] Quatrini L Ricci B Ciancaglini C Tumino N Moretta L. Regulation of the immune system development by glucocorticoids and sex hormones. Front Immunol. (2021) 12:672853. doi: 10.3389/fimmu.2021.672853 34248954 PMC8260976

[8] Mariotti A Mawhinney M. Endocrinology of sex steroid hormones and cell dynamics in the periodontium. Periodontol 2000. (2013) 61:69–88. doi: 10.1111/j.1600-0757.2011.00424.x 23240944

[9] Cornejo Ulloa P Krom BP van der Veen MH. Sex steroid hormones as a balancing factor in oral host microbiome interactions. Front Cell Infection Microbiol. (2021) 11. doi: 10.3389/fcimb.2021.714229 PMC8511811 34660339

[10] Diotel N Charlier TD Lefebvre d’Hellencourt C Couret D Trudeau VL Nicolau JC. Steroid transport, local synthesis, and signaling within the brain: roles in neurogenesis, neuroprotection, and sexual behaviors. Front Neurosci. (2018) 12:84. doi: 10.3389/fnins.2018.00084 29515356 PMC5826223

[11] Simpson ER Boyd GS. The cholesterol side-chain cleavage system of the adrenal cortex: a mixed-function oxidase. Biochem Biophys Res Commun. (1966) 24:10–7. doi: 10.1016/0006-291X(66)90402-5 5966391

[12] Li L Zirkin BR Papadopoulos V. Leydig Cell Androgen Synthesis. In: Skinner MK, editor. Encyclopedia of Reproduction, 2nd ed. Academic Press, Oxford (2018). p. 215–21.

[13] Hu J Zhang Z Shen W-J Azhar S. Cellular cholesterol delivery, intracellular processing and utilization for biosynthesis of steroid hormones. Nutr Metab. (2010) 7:47. doi: 10.1186/1743-7075-7-47 PMC2890697 20515451

[14] Miller WL Auchus RJ. The molecular biology, biochemistry, and physiology of human steroidogenesis and its disorders. Endocrine Rev. (2011) 32:81–151. doi: 10.1210/er.2010-0013 21051590 PMC3365799

[15] Compagnone NA Mellon SH. Neurosteroids: biosynthesis and function of these novel neuromodulators. Front Neuroendocrinol. (2000) 21:1–56. doi: 10.1006/frne.1999.0188 10662535

[16] Nikolakis G Stratakis CA Kanaki T Slominski A Zouboulis CC. Skin steroidogenesis in health and disease. Rev Endocrine Metab Disord. (2016) 17:247–58. doi: 10.1007/s11154-016-9390-z 27761789

[17] Li J Papadopoulos V Vihma V. Steroid biosynthesis in adipose tissue. Steroids. (2015) 103:89–104. doi: 10.1016/j.steroids.2015.03.016 25846979

[18] Honour JW. Chapter 1.3 - Steroid biosynthesis. In: Honour JW, editor. Steroids in the Laboratory and Clinical Practice. University College London, United Kingdom: Elsevier (2023). p. 63–92.

[19] Saldanha CJ Remage-Healey L Schlinger BA. Synaptocrine signaling: steroid synthesis and action at the synapse. Endocr Rev. (2011) 32:532–49. doi: 10.1210/er.2011-0004 PMC3369574 21622487

[20] Pillerová M Borbélyová V Hodosy J Riljak V Renczés E Frick KM. On the role of sex steroids in biological functions by classical and non-classical pathways. update Front Neuroendocrinol. (2021) 62:100926. doi: 10.1016/j.yfrne.2021.100926 34089761 PMC8523217

[21] Narinx N David K Walravens J Vermeersch P Claessens F Fiers T. Role of sex hormone-binding globulin in the free hormone hypothesis and the relevance of free testosterone in androgen physiology. Cell Mol Life Sci. (2022) 79:543. doi: 10.1007/s00018-022-04562-1 36205798 PMC11803068

[22] Southren AL Rappaport SC Gordon GG Vittek J. Specific 5 alpha-dihydrotestosterone receptors in human gingiva. J Clin Endocrinol Metab. (1978) 47:1378–82. doi: 10.1210/jcem-47-6-1378 263357

[23] Vittek J Hernandez MR Wenk EJ Rappaport SC Southren AL. Specific estrogen receptors in human gingiva. J Clin Endocrinol Metab. (1982) 54:608–12. doi: 10.1210/jcem-54-3-608 7035487

[24] Parkar M Newman HN Olsen I. Polymerase chain reaction analysis of oestrogen and androgen receptor expression in human gingival and periodontal tissue. Arch Oral Biol. (1996) 41:979–83. doi: 10.1016/S0003-9969(96)00053-2 9031705

[25] Tricco AC Lillie E Zarin W O’Brien KK Colquhoun H Levac D. PRISMA extension for scoping reviews (PRISMA-scR): checklist and explanation. Ann Intern Med. (2018) 169:467–73. doi: 10.7326/M18-0850 30178033

[26] Ouzzani M Hammady H Fedorowicz Z Elmagarmid A. Rayyan—a web and mobile app for systematic reviews. Systematic Rev. (2016) 5:210. doi: 10.1186/s13643-016-0384-4 PMC5139140 27919275

[27] The Endnote Team. Endnote. Philadelphia, PA: Calrivate (2013).

[28] Lobbestael G. DedupEndNote. 0.9.7e ed2023.

[29] Raju K Berens L. Periodontology and pregnancy: An overview of biomedical and epidemiological evidence. Periodontol 2000. (2021) 87:132–42. doi: 10.1111/prd.12394 34463990

[30] Beck JD Papapanou PN Philips KH Offenbacher S. Periodontal medicine: 100 years of progress. J Dent Res. (2019) 98:1053–62. doi: 10.1177/0022034519846113 31429666

[31] Yu T Volponi AA Babb R An Z Sharpe PT. Chapter Eight - Stem Cells in Tooth Development, Growth, Repair, and Regeneration. In: Chai Y, editor. Current Topics in Developmental Biology, vol. 115. London, United Kingdom: Academic Press (2015). p. 187–212. 10.1016/bs.ctdb.2015.07.010 26589926

[32] Liu J Zhao Z Ruan J Weir MD Ma T Ren K. Stem cells in the periodontal ligament differentiated into osteogenic, fibrogenic and cementogenic lineages for the regeneration of the periodontal complex. J Dentistry. (2020) 92:103259. doi: 10.1016/j.jdent.2019.103259 31809792

[33] Posnick JC. 6 - Periodontal Considerations in the Evaluation and Treatment of Dentofacial Deformities. In: Posnick JC, editor. Orthognathic Surgery. W.B. Saunders, St. Louis (2014). p. 171–208.

[34] Hughes FJ. Chapter 34 - Periodontium and Periodontal Disease. In: Vishwakarma A Sharpe P Shi S Ramalingam M, editors. Stem Cell Biology and Tissue Engineering in Dental Sciences. Academic Press, Boston (2015). p. 433–44.

[35] Kawahara K Shimazu A. Expression and intracellular localization of progesterone receptors in cultured human gingival fibroblasts. J Periodontal Res. (2003) 38:242–6. doi: 10.1034/j.1600-0765.2003.00654.x 12753360

[36] Coletta RD Reynolds MA Martelli-Junior H Graner E Almeida OP Sauk JJ. Testosterone stimulates proliferation and inhibits interleukin-6 production of normal and hereditary gingival fibromatosis fibroblasts. Oral Microbiol Immunol. (2002) 17:186–92. doi: 10.1034/j.1399-302X.2002.170309.x 12030972

[37] Gornstein RA Lapp CA Bustos-Valdes SM Zamorano P. Androgens modulate interleukin-6 production by gingival fibroblasts in vitro. J Periodontol. (1999) 70:604–9. doi: 10.1902/jop.1999.70.6.604 10397515

[38] Nebel D Bratthall G Ekblad E Norderyd O Nilsson BO. Estrogen regulates DNA synthesis in human gingival epithelial cells displaying strong estrogen receptor β immunoreactivity. J Periodontal Res. (2011) 46:622–8. doi: 10.1111/j.1600-0765.2011.01382.x 21615412

[39] Välimaa H Savolainen S Soukka T Silvoniemi P Mäkelä S Kujari H. Estrogen receptor-beta is the predominant estrogen receptor subtype in human oral epithelium and salivary glands. J endocrinol. (2004) 180:55–62. doi: 10.1677/joe.0.1800055 14709144

[40] ElAttar TM Roth GD Hugoson A. Comparative metabolism of 4- 14 C-progesterone in normal and chronically inflamed human gingival tissue. J Periodontal Res. (1973) 8:79–85. doi: 10.1111/j.1600-0765.1973.tb00746.x 4267949

[41] Ojanotko-Harri A. Metabolism of progesterone by healthy and inflamed human gingiva in vitro. J Steroid Biochem. (1985) 23:1031–5. doi: 10.1016/0022-4731(85)90063-9 4094411

[42] Tilakaratne A Soory M. Androgen metabolism in response to oestradiol-17beta and progesterone in human gingival fibroblasts (HGF) in culture. J Clin periodontol. (1999) 26:723–31. doi: 10.1034/j.1600-051X.1999.t01-4-261101.x 10589808

[43] Tilakaratne A Soory M. Modulation of androgen metabolism by estradiol-17beta and progesterone, alone and in combination, in human gingival fibroblasts in culture. J periodontol. (1999) 70:1017–25. doi: 10.1902/jop.1999.70.9.1017 10505804

[44] ElAttar TM. The in *vitro* conversion of male sex steroid, (1,2-3-H)-androstenedione in normal and inflamed human gingiva. Arch Oral Biol. (1974) 19:1185–90. doi: 10.1016/0003-9969(74)90250-7 4531881

[45] Vittek J Rappaport SC Gordon GG Munnangi PR Southren AL. Concentration of circulating hormones and metabolism of androgens by human gingiva. J periodontol. (1979) 50:254–64. doi: 10.1902/jop.1979.50.5.254 287781

[46] Ojanotko A Nienstedt W Harri MP. Metabolism of testosterone by human healthy and inflamed gingiva in vitro. Arch Oral Biol. (1980) 25:481–4. doi: 10.1016/0003-9969(80)90055-2 6935996

[47] ElAttar TM Hugoson A. The in *vitro* conversion of female sex steroid, oestrone, in normal and inflamed human gingiva. Arch Oral Biol. (1974) 19:425–9. doi: 10.1016/0003-9969(74)90147-2 4531294

[48] Mariotti A. Estrogen and extracellular matrix influence human gingival fibroblast proliferation and protein production. J periodontol. (2005) 76:1391–7. doi: 10.1902/jop.2005.76.8.1391 16101374

[49] Handajani J Effendi N Sosroseno W. Correlation between salivary estrogen levels and oral epithelial cytokeratin 5 expression. F1000Res. (2020) 9:186. doi: 10.12688/f1000research 32399205 PMC7194496

[50] Yokoyama M Hinode D Masuda K Yoshioka M Grenier D. Effect of female sex hormones on Campylobacter rectus and human gingival fibroblasts. Oral Microbiol Immunol. (2005) 20:239–43. doi: 10.1111/j.1399-302X.2005.00222.x 15943769

[51] Ostad SN Motahhary P Beshkar M Ghahremani MH. 17β-estradiol and progesterone upregulate cyclooxygenase-2 expression in the human gingival fibroblasts. Daru. (2006) 14:190–6.

[52] Parkar M Tabona P Newman H Olsen I. IL-6 expression by oral fibroblasts is regulated by androgen. Cytokine. (1998) 10:613–9. doi: 10.1006/cyto.1998.0336 9722934

[53] Konermann A Winter J Novak N Allam JP Jäger A. Verification of IL-17A and IL-17F in oral tissues and modulation of their expression pattern by steroid hormones. Cell Immunol. (2013) 285:133–40. doi: 10.1016/j.cellimm.2013.10.004 24185279

[54] ElAttar TM Lin HS Tira DE. Testosterone inhibits prostaglandin formation by human gingival connective tissue: relationship to 14C-arachidonic acid metabolism. Prostaglandins Leukot Med. (1982) 9:25–34. doi: 10.1016/0262-1746(82)90069-5 6813875

[55] Shu L Guan SM Fu SM Guo T Cao M Ding Y. Estrogen modulates cytokine expression in human periodontal ligament cells. J Dental Res. (2008) 87:142–7. doi: 10.1177/154405910808700214 18218840

[56] Lewko WM Anderson A. Estrogen receptors and growth response in cultured human periodontal ligament cells. Life Sci. (1986) 39:1201–6. doi: 10.1016/0024-3205(86)90352-8 3747726

[57] Yuan G Cai C Dai J Liu Y Zhang R Dai Y. Progesterone modulates the proliferation and differentiation of human periodontal ligament cells. Calcified Tissue Int. (2010) 87:158–67. doi: 10.1007/s00223-010-9377-9 20532879

[58] Morishita M Shimazu A Iwamoto Y. Analysis of oestrogen receptor mRNA by reverse transcriptase-polymerase chain reaction in human periodontal ligament cells. Arch Oral Biol. (1999) 44:781–3. doi: 10.1016/S0003-9969(99)00063-1 10471162

[59] Jönsson D Andersson G Ekblad E Liang M Bratthall G Nilsson BO. Immunocytochemical demonstration of estrogen receptor beta in human periodontal ligament cells. Arch Oral Biol. (2004) 49:85–8. doi: 10.1016/s0003-9969(03)00198-5 14693201

[60] Jönsson D Nilsson J Odenlund M Bratthall G Broman J Ekblad E. Demonstration of mitochondrial oestrogen receptor beta and oestrogen-induced attenuation of cytochrome c oxidase subunit I expression in human periodontal ligament cells. Arch Oral Biol. (2007) 52:669–76. doi: 10.1016/j.archoralbio.2006.12.009 17223066

[61] Liang L Yu JF Wang Y Ding Y. Estrogen regulates expression of osteoprotegerin and RANKL in human periodontal ligament cells through estrogen receptor beta. J periodontol. (2008) 79:1745–51. doi: 10.1902/jop.2008.070437 18771377

[62] Mamalis A Markopoulou C Lagou A Vrotsos I. Oestrogen regulates proliferation, osteoblastic differentiation, collagen synthesis and periostin gene expression in human periodontal ligament cells through oestrogen receptor beta. Arch Oral Biol. (2011) 56:446–55. doi: 10.1016/j.archoralbio.2010.11.001 21130420

[63] Tang X Meng H Han J Zhang L Hou J Zhang F. Up-regulation of estrogen receptor-beta expression during osteogenic differentiation of human periodontal ligament cells. J periodontal Res. (2008) 43:311–21. doi: 10.1111/j.1600-0765.2007.01031.x 18004992

[64] Cao M Shu L Li J Su J Zhang W Wang Q. The expression of estrogen receptors and the effects of estrogen on human periodontal ligament cells. Methods Find Exp Clin Pharmacol. (2007) 29:329–35. doi: 10.1358/mf.2007.29.5.1117560 17805434

[65] Cai C Yuan GJ Huang Y Yang N Chen X Wen L. Estrogen-related receptor α is involved in the osteogenic differentiation of mesenchymal stem cells isolated from human periodontal ligaments. Int J Mol Med. (2013) 31:1195–201. doi: 10.3892/ijmm.2013.1305 23525223

[66] Pan F Zhang R Wang G Ding Y. Oestrogen receptors are involved in the osteogenic differentiation of periodontal ligament stem cells. Biosci Rep. (2011) 31:117–24. doi: 10.1042/BSR20100029 20524935

[67] Vittek J Rappaport SC Gordon GG Hagedoorn J Southren AL. Metabolism of androgens by human periodontal ligament. J Dental Res. (1982) 61:1153–7. doi: 10.1177/00220345820610100801 6956595

[68] Jönsson D Wahlin Å Idvall I Johnsson I Bratthall G Nilsson B-O. Differential effects of estrogen on DNA synthesis in human periodontal ligament and breast cancer cells. J Periodontal Res. (2005) 40:401–6. doi: 10.1111/j.1600-0765.2005.00821.x 16105093

[69] Wattanaroonwong N Schoenmaker T de Vries TJ Everts V. Oestrogen inhibits osteoclast formation induced by periodontal ligament fibroblasts. Arch Oral Biol. (2011) 56:212–9. doi: 10.1016/j.archoralbio.2010.10.004 21035111

[70] Liang L Yu JF Wang Y Wang G Ding Y. Effect of estrogen receptor beta on the osteoblastic differentiation function of human periodontal ligament cells. Arch Oral Biol. (2008) 53:553–7. doi: 10.1016/j.archoralbio.2007.12.011 18261710

[71] Morishita M Yamamura T Bachchu MA Shimazu A Iwamoto Y. The effects of oestrogen on osteocalcin production by human periodontal ligament cells. Arch Oral Biol. (1998) 43:329–33. doi: 10.1016/S0003-9969(97)00114-3 9839709

[72] Morishita M Yamamura T Shimazu A Bachchu AH Iwamoto Y. Estradiol enhances the production of mineralized nodules by human periodontal ligament cells. J Clin Periodontol. (1999) 11:748–51. doi: 10.1034/j.1600-051X.1999.t01-7-261101.x 10589811

[73] Jiang B Xu J Zhou Y Mao J Guan G Xu X. Estrogen enhances osteogenic differentiation of human periodontal ligament stem cells by activating the wnt/β-catenin signaling pathway. J craniofacial surgery. (2020) 31:583–7. doi: 10.1097/SCS.0000000000006226 31977705

[74] Son YB Kang YH Lee HJ Jang SJ Bharti D Lee SL. Evaluation of odonto/osteogenic differentiation potential from different regions derived dental tissue stem cells and effect of 17β-estradiol on efficiency. BMC Oral Health. (2021) 21:15. doi: 10.1186/s12903-020-01366-2 33413268 PMC7792121

[75] Liao J Zhou Z Huang L Li Y Li J Zou S. 17β-estradiol regulates the differentiation of cementoblasts via Notch signaling cascade. Biochem Biophys Res Commun. (2016) 477:109–14. doi: 10.1016/j.bbrc.2016.06.028 27289020

[76] Nuñez J Sanz-Blasco S Vignoletti F Muñoz F Caffesse RG Sanz M. 17beta-estradiol promotes cementoblast proliferation and cementum formation in experimental periodontitis. J Periodontol. (2010) 7:1064–74. doi: 10.1902/jop.2010.090678 20214440

[77] MacNamara P O’Shaughnessy C Manduca P Loughrey HC. Progesterone receptors are expressed in human osteoblast-like cell lines and in primary human osteoblast cultures. Calcif Tissue Int. (1995) 57:436–41. doi: 10.1007/BF00301947 8581876

[78] Eriksen EF Colvard DS Berg NJ Graham ML Mann KG Spelsberg TC. Evidence of estrogen receptors in normal human osteoblast-like cells. Science. (1988) 241:84–6. doi: 10.1126/science.3388021 3388021

[79] Colvard DS Eriksen EF Keeting PE Wilson EM Lubahn DB French FS. Identification of androgen receptors in normal human osteoblast-like cells. Proc Natl Acad Sci. (1989) 86:854–7. doi: 10.1073/pnas.86.3.854 PMC286576 2915981

[80] Imai Y Youn M-Y Inoue K Takada I Kouzmenko A Kato S. Nuclear receptors in bone physiology and diseases. Physiol Rev. (2013) 93:481–523. doi: 10.1152/physrev.00008.2012 23589826 PMC3768103

[81] Khalid AB Krum SA. Estrogen receptors alpha and beta in bone. Bone. (2016) 87:130–5. doi: 10.1016/j.bone.2016.03.016 PMC5336249 27072516

[82] ElAttar TM. Metabolism of progesterone -7α-(3) H in vitro in human gingiva with periodontitis. J periodontol. (1971) 42:721–5. doi: 10.1902/jop.1971.42.11.721 5288567

[83] Kasasa SC Soory M. The effect of interleukin-1 (IL-1) on androgen metabolism in human gingival tissue (HGT) and periodontal ligament (PDL). J Clin Periodontol. (1996) 23:419–24. doi: 10.1111/j.1600-051X.1996.tb00568.x 8783045

[84] Kasasa SC Soory M. The effect of PDGF, TGF-beta and IGF in combination on androgen metabolism by fibroblasts. J Clin Periodontol. (1998) 8:640–6. doi: 10.1111/j.1600-051X.1998.tb02500.x 9722268

[85] Kasasa SC Soory M. The combined effects of TGF-beta, IGF and PDGF on 5alpha-reductase activity on androgen substrates in human gingival tissue. Inflammopharmacology. (1998) 6:223–34. doi: 10.1007/s10787-998-0021-5 17657621

[86] Soory M Gower DB. The influence of prostaglandins on steroid conversions by human gingival fibroblasts. J periodontal Res. (1998) 33:439–47. doi: 10.1111/j.1600-0765.1998.tb02342.x 9879516

[87] Soory M Tilakaratne A. Modulation of androgen metabolism by phenytoin, oestradiol and tamoxifen in human gingival fibroblasts. J Clin periodontol. (2003) 30:556–61. doi: 10.1034/j.1600-051X.2003.00302.x 12795795

[88] Cauley JA. Estrogen and bone health in men and women. Steroids. (2015) 99:11–5. doi: 10.1016/j.steroids.2014.12.010 25555470

[89] Purohit A Flanagan AM Reed MJ. Estrogen synthesis by osteoblast cell lines. Endocrinology. (1992) 131:2027–9. doi: 10.1210/endo.131.4.1396346 1396346

[90] Janssen JM Bland R Hewison M Coughtrie MW Sharp S Arts J. Estradiol formation by human osteoblasts via multiple pathways: relation with osteoblast function. J Cell Biochem. (1999) 75:528–37. doi: 10.1002/(ISSN)1097-4644 10536374

[91] Khosla S Oursler MJ Monroe DG. Estrogen and the skeleton. Trends Endocrinol Metab. (2012) 23:576–81. doi: 10.1016/j.tem.2012.03.008 PMC3424385 22595550

[92] Lapp CA Thomas ME Lewis JB. Modulation by progesterone of interleukin-6 production by gingival fibroblasts. J Periodontol. (1995) 66:279–84. doi: 10.1902/jop.1995.66.4.279 7782982

[93] Maloney JP Gao L. Proinflammatory cytokines increase vascular endothelial growth factor expression in alveolar epithelial cells. Mediators Inflamm. (2015) 2015:387842. doi: 10.1155/2015/387842 26424968 PMC4573992

[94] Huang SP Wu MS Shun CT Wang HP Lin MT Kuo ML. Interleukin-6 increases vascular endothelial growth factor and angiogenesis in gastric carcinoma. J BioMed Sci. (2004) 11:517–27. doi: 10.1007/BF02256101 15153787

[95] Lapp CA Lapp DF. Analysis of interleukin-activated human gingival fibroblasts: modulation of chemokine responses by female hormones. J Periodontol. (2005) 76:803–12. doi: 10.1902/jop.2005.76.5.803 15898942

[96] Nebel D Jönsson D Norderyd O Bratthall G Nilsson BO. Differential regulation of chemokine expression by estrogen in human periodontal ligament cells. J periodontal Res. (2010) 45:796–802. doi: 10.1111/jre.2010.45.issue-6 20701669

[97] Kasasa SC Soory M. The response of human gingival fibroblasts to interleukin-1 in their metabolic conversion of two androgenic substrates. Arch Oral Biol. (1995) 40:979–81. doi: 10.1016/0003-9969(95)00058-W 8526810

[98] Arid J Oliveira DB Evangelista SS Vasconcelos KRF Dutra ALT de Oliveira SS. Oestrogen receptor alpha, growth hormone receptor, and developmental defect of enamel. Int J paediatric dentistry. (2019) 29:29–35. doi: 10.1111/ipd.12434 30341791

[99] Weber ML Hsin HY Kalay E Brožková D Shimizu T Bayram M. Role of estrogen related receptor beta (ESRRB) in DFN35B hearing impairment and dental decay. BMC Med Genet. (2014) 15:81. doi: 10.1186/1471-2350-15-81 25023176 PMC4112727

[100] Dalledone M Cunha AS Ramazzotto LA Pecharki GD Nelson-Filho P Scariot R. Estrogen receptor gene is associated with dental fluorosis in Brazilian children. Clin Oral Investig. (2019) 23:3565–70. doi: 10.1007/s00784-018-2778-2 30539292

[101] Ba Y Zhang H Wang G Wen S Yang Y Zhu J. Association of dental fluorosis with polymorphisms of estrogen receptor gene in Chinese children. Biol Trace Elem Res. (2011) 143:87–96. doi: 10.1007/s12011-010-8848-1 20852966

[102] Whitaker SB Singh BB Weller RN Bath KR Loushine RJ. Sex hormone receptor status of the dental pulp and lesions of pulpal origin. Oral surgery Oral med Oral pathol Oral radiol endodontics. (1999) 87:233–7. doi: 10.1016/S1079-2104(99)70278-7 10052381

[103] Inaba T Kobayashi T Tsutsui TW Ogawa M Uchida M Tsutsui T. Expression status of mRNA for sex hormone receptors in human dental pulp cells and the response to sex hormones in the cells. Arch Oral Biol. (2013) 58:943–50. doi: 10.1016/j.archoralbio.2013.02.001 23490353

[104] Dale JB Sarich SL Bretz TM Hatton JF Zachow RJ. Hormonal regulation of androgen receptor messenger ribonucleic acid expression in human tooth pulp. J Dental Res. (2002) 81:360–5. doi: 10.1177/154405910208100514 12097452

[105] Alhodhodi A Alkharobi H Humphries M Alkhafaji H El-Gendy R Feichtinger G. Oestrogen receptor β (ERβ) regulates osteogenic differentiation of human dental pulp cells. J Steroid Biochem Mol Biol. (2017) 174:296–302. doi: 10.1016/j.jsbmb.2017.10.012 29031686

[106] Jukić S Prpić-Mehicić G Talan-Hranilovć J Miletić I Segović S Anić I. Estrogen receptors in human pulp tissue. Oral surgery Oral med Oral pathol Oral radiol endodontics. (2003) 95:340–4. doi: 10.1067/moe.2003.9 12627107

[107] Hietala EL Larmas M Salo T. Localization of estrogen-receptor-related antigen in human odontoblasts. J Dental Res. (1998) 77:1384–7. doi: 10.1177/00220345980770060201 9649166

[108] Wang Y Zheng Y Wang Z Li J Wang Z Zhang G. 10(-7) m 17β-oestradiol enhances odonto/osteogenic potency of human dental pulp stem cells by activation of the NF-κB pathway. Cell Prolif. (2013) 46:677–84. doi: 10.1111/cpr.12071 PMC4065368 24152244

[109] Woo SM Seong KJ Oh SJ Park HJ Kim SH Kim WJ. 17β-Estradiol induces odontoblastic differentiation via activation of the c-Src/MAPK pathway in human dental pulp cells. Biochem Cell Biol = Biochimie biologie cellulaire. (2015) 93:587–95. doi: 10.1139/bcb-2015-0036 26393498

[110] Wang Y Lu Y Li Z Zhou Y Gu Y Pang X. Oestrogen receptor α regulates the odonto/osteogenic differentiation of stem cells from apical papilla via ERK and JNK MAPK pathways. Cell Prolif. (2018) 51:e12485. doi: 10.1111/cpr.12485 30069950 PMC6528913

[111] Li Y Yan M Wang Z Zheng Y Li J Ma S. 17beta-estradiol promotes the odonto/osteogenic differentiation of stem cells from apical papilla via mitogen-activated protein kinase pathway. Stem Cell Res Ther. (2014) 5:125. doi: 10.1186/scrt515 25403930 PMC4446088

[112] Manokawinchoke J Ritprajak P Osathanon T Pavasant P. Estradiol induces osteoprotegerin expression by human dental pulp cells. Odontology. (2016) 104:10–8. doi: 10.1007/s10266-014-0178-x 25255977

[113] Jedeon K Loiodice S Salhi K Le Normand M Houari S Chaloyard J. Androgen receptor involvement in rat amelogenesis: an additional way for endocrine-disrupting chemicals to affect enamel synthesis. Endocrinology. (2016) 157:4287–96. doi: 10.1210/en.2016-1342 27684650

[114] Ferrer VL Maeda T Kawano Y. Characteristic distribution of immunoreaction for estrogen receptor alpha in rat ameloblasts. Anat Rec A Discovery Mol Cell Evol Biol. (2005) 284:529–36. doi: 10.1002/(ISSN)1552-4892 15803481

[115] Cheng CH Chen LR Chen KH. Osteoporosis due to hormone imbalance: an overview of the effects of estrogen deficiency and glucocorticoid overuse on bone turnover. Int J Mol Sci. (2022) 23:1376. doi: 10.3390/ijms23031376 35163300 PMC8836058

[116] Şenel S. An overview of physical, microbiological and immune barriers of oral mucosa. Int J Mol Sci. (2021) 22:7821. doi: 10.3390/ijms22157821 34360589 PMC8346143

[117] Winning TA Townsend GC. Oral mucosal embryology and histology. Clinics Dermatol. (2000) 18:499–511. doi: 10.1016/S0738-081X(00)00140-1 11134845

[118] Ojanotko-Harri A Forssell H Laine M Hurttia H Bläuer M Tuohimaa P. Immunohistochemical detection of androgen receptors in human oral mucosa. Arch Oral Biol. (1992) 37:511–4. doi: 10.1016/0003-9969(92)90108-K 1637265

[119] Leimola-Virtanen R Salo T Toikkanen S Pulkkinen J Syrjänen S. Expression of estrogen receptor (ER) in oral mucosa and salivary glands. Maturitas. (2000) 36:131–7. doi: 10.1016/S0378-5122(00)00138-9 11006500

[120] Engeland CG Bosch JA Cacioppo JT Marucha PT. Mucosal wound healing: the roles of age and sex. Arch Surg. (2006) 141:1193–7; discussion 8. doi: 10.1001/archsurg.141.12.1193 17178961

[121] Engeland CG Sabzehei B Marucha PT. Sex hormones and mucosal wound healing. Brain Behav Immun. (2009) 7:629–35. doi: 10.1016/j.bbi.2008.12.001 PMC2746088 19111925

[122] Wu T Tang C Chen Y Yong X Liu Z Jiang L. Regulatory effect of 17β-estradiol on the expression of β-defensin-2 and proinflammatory cytokines in human oral epithelial cells. J Oral Pathol Med. (2020) 49:365–72. doi: 10.1111/jop.13016 32176389

[123] Taga T Ito K Takamatsu K Ogawa M Funayama S Inoue M. Menopausal symptoms are associated with oral sensory complaints in perimenopausal women: an observational study. BMC Womens Health. (2021) 21:262. doi: 10.1186/s12905-021-01401-6 34193118 PMC8243452

[124] Oudhoff MJ van den Keijbus PA Kroeze KL Nazmi K Gibbs S Bolscher JG. Histatins enhance wound closure with oral and non-oral cells. J Dent Res. (2009) 88:846–50. doi: 10.1177/0022034509342951 19767583

[125] Horng HC Chang WH Yeh CC Huang BS Chang CP Chen YJ. Estrogen effects on wound healing. Int J Mol Sci. (2017) 18:2325. doi: 10.3390/ijms18112325 29099810 PMC5713294

[126] Tuo B Wen G Wei J Liu X Wang X Zhang Y. Estrogen regulation of duodenal bicarbonate secretion and sex-specific protection of human duodenum. Gastroenterology. (2011) 141:854–63. doi: 10.1053/j.gastro.2011.05.044 PMC3163800 21699784

[127] Moutsopoulos NM Konkel JE. Tissue-specific immunity at the oral mucosal barrier. Trends Immunol. (2018) 39:276–87. doi: 10.1016/j.it.2017.08.005 PMC5843496 28923364

[128] Belkaid Y Harrison OJ. Homeostatic immunity and the microbiota. Immunity. (2017) 46:562–76. doi: 10.1016/j.immuni.2017.04.008 PMC5604871 28423337

[129] Dutzan N Abusleme L Bridgeman H Greenwell-Wild T Zangerle-Murray T Fife ME. On-going mechanical damage from mastication drives homeostatic th17 cell responses at the oral barrier. Immunity. (2017) 46:133–47. doi: 10.1016/j.immuni.2016.12.010 PMC5263257 28087239

[130] Shang L Deng D Roffel S Gibbs S. Differential influence of Streptococcus mitis on host response to metals in reconstructed human skin and oral mucosa. Contact Dermatitis. (2020) 83:347–60. doi: 10.1111/cod.13668 PMC7693211 32677222

[131] de Paula F Teshima THN Hsieh R Souza MM Nico MMS Lourenco SV. Overview of human salivary glands: highlights of morphology and developing processes. Anat Rec (Hoboken). (2017) 300:1180–8. doi: 10.1002/ar.23569 28192873

[132] Shafer WG Muhler JC. Effect of gonadectomy and sex hormones on the structure of the rat salivary glands. J Dental Res. (1953) 32:262–8. doi: 10.1177/00220345530320021501 13052779

[133] Clark PG Shafer WG Muhler JC. Effect of hormones on structure and proteolytic activity of salivary glands. J Dental Res. (1957) 36:403–8. doi: 10.1177/00220345570360031301 13428898

[134] Hosoi K Kobayashi S Hiramatsu M Minami N Ueha T. Androgenic regulation of N-acetyl beta-glucosaminidase activity in the submandibular glands of mice. J Biochem. (1979) 6:1483–8. doi: 10.1093/oxfordjournals.jbchem.a132476 457644

[135] Booth WD. Sexual dimorphism involving steroidal pheromones and their binding protein in the submaxillary salivary gland of the Göttingen miniature pig. J Endocrinol. (1984) 100:195–202. doi: 10.1677/joe.0.1000195 6537966

[136] Laine M Bläuer M Ylikomi T Tuohimaa P Aitasalo K Happonen RP. Immunohistochemical demonstration of androgen receptors in human salivary glands. Arch Oral Biol. (1993) 38:299–302. doi: 10.1016/0003-9969(93)90136-A 8517801

[137] Whitaker SB Vigneswaran N Singh BB. Androgen receptor status of the oral sebaceous glands. Am J Dermatopathol. (1997) 19:415–8. doi: 10.1097/00000372-199708000-00018 9261481

[138] Tsinti M Kassi E Korkolopoulou P Kapsogeorgou E Moutsatsou P Patsouris E. Functional estrogen receptors alpha and beta are expressed in normal human salivary gland epithelium and apparently mediate immunomodulatory effects. Eur J Oral Sci. (2009) 117:498–505. doi: 10.1111/j.1600-0722.2009.00659.x 19758244

[139] Blom T Ojanotko-Harri A Laine M Huhtaniemi I. Metabolism of progesterone and testosterone in human parotid and submandibular salivary glands in vitro. J Steroid Biochem Mol Biol. (1993) 44:69–76. doi: 10.1016/0960-0760(93)90153-N 8424895

[140] Shiau HJ Reynolds MA. Sex differences in destructive periodontal disease: exploring the biologic basis. J Periodontol. (2010) 81:1505–17. doi: 10.1902/jop.2010.100045 20594052

[141] Steffens JP Wang X Starr JR Spolidorio LC Van Dyke TE Kantarci A. Associations between sex hormone levels and periodontitis in men: results from NHANES III. J Periodontol. (2015) 86:1116–25. doi: 10.1902/jop.2015.140530 26062840

[142] Saranya R Chandini R Mohideen K PN A Subramani V Balasubramaniam M. Expression of sex hormones in oral squamous cell carcinoma: A systematic review on immunohistochemical studies. Cureus. (2022) 14:e25384. doi: 10.7759/cureus.25384 35765387 PMC9233754

[143] Jussila P Kiviahde H Näpänkangas R Päkkilä J Pesonen P Sipilä K. Prevalence of temporomandibular disorders in the northern Finland birth cohort 1966. J Oral Facial Pain Headache. (2017) 31:159–64. doi: 10.11607/ofph.1773 28437513

[144] Qin B Wang J Yang Z Yang M Ma N Huang F. Epidemiology of primary Sjögren’s syndrome: a systematic review and meta-analysis. Ann Rheum Dis. (2015) 74:1983–9. doi: 10.1136/annrheumdis-2014-205375 24938285

[145] Wu S Zhang W Yan J Noma N Young A Yan Z. Worldwide prevalence estimates of burning mouth syndrome: A systematic review and meta-analysis. Oral Dis. (2022) 28:1431–40. doi: 10.1111/odi.13868 33818878

[146] Vitali C Bombardieri S Jonsson R Moutsopoulos HM Alexander EL Carsons SE. Classification criteria for Sjögren’s syndrome: a revised version of the European criteria proposed by the American-European Consensus Group. Ann Rheum Dis. (2002) 61:554–8. doi: 10.1136/ard.61.6.554 PMC1754137 12006334

[147] Forsblad-d’Elia H Carlsten H Labrie F Konttinen YT Ohlsson C. Low serum levels of sex steroids are associated with disease characteristics in primary sjogren’s syndrome; supplementation with dehydroepiandrosterone restores the concentrations. J Clin Endocrinol Metab. (2009) 94:2044–51. doi: 10.1210/jc.2009-0106 19318446

[148] das Neves de Araújo Lima E Barbosa NG Dos Santos AC Araújo Moura Lemos TM de Souza CM Trevilatto PC. Comparative analysis of psychological, hormonal, and genetic factors between burning mouth syndrome and secondary oral burning. Pain Med. (2016) 9:1602–11. doi: 10.1093/pm/pnv087 26849950

[149] Robinson JL Johnson PM Kister K Yin MT Chen J Wadhwa S. Estrogen signaling impacts temporomandibular joint and periodontal disease pathology. Odontology. (2020) 108:153–65. doi: 10.1007/s10266-019-00439-1 PMC7192637 31270648

[150] Ribeiro-Dasilva MC Peres Line SR Leme Godoy dos Santos MC Arthuri MT Hou W Fillingim RB. Estrogen receptor-alpha polymorphisms and predisposition to TMJ disorder. J Pain. (2009) 10:527–33. doi: 10.1016/j.jpain.2008.11.012 PMC2749669 19411060

[151] Gay L Melenotte C Lakbar I Mezouar S Devaux C Raoult D. Sexual dimorphism and gender in infectious diseases. Front Immunol. (2021) 12. doi: 10.3389/fimmu.2021.698121 PMC8339590 34367158

[152] Chakraborty S Pramanik J Mahata B. Revisiting steroidogenesis and its role in immune regulation with the advanced tools and technologies. Genes Immunity. (2021) 22:125–40. doi: 10.1038/s41435-021-00139-3 PMC8277576 34127827

[153] Miller WL Gucev ZS. Chapter 3I - Disorders in the Initial Steps in Steroidogenesis. In: New MI Lekarev O Parsa A Yuen TT O’Malley BW Hammer GD, editors. Genetic Steroid Disorders. Academic Press, San Diego (2014). p. 145–64.

[154] Di Donato V Iacobelli V Schiavi MC Colagiovanni V Pecorella I Palaia I. Impact of hormone receptor status and ki-67 expression on disease-free survival in patients affected by high-risk endometrial cancer. Int J Gynecologic Cancer. (2018) 28:505–13. doi: 10.1097/IGC.0000000000001191 29465508

[155] La Greca A Bellora N Le Dily F Jara R Nacht AS Quilez Oliete J. Chromatin topology defines estradiol-primed progesterone receptor and PAX2 binding in endometrial cancer cells. eLife. (2022) 11:e66034. doi: 10.7554/eLife.66034 35018885 PMC8887898

[156] Sathish V Martin YN Prakash YS. Sex steroid signaling: implications for lung diseases. Pharmacol Ther. (2015) 150:94–108. doi: 10.1016/j.pharmthera.2015.01.007 25595323 PMC4523383

[157] Mor G Yue W Santen RJ Gutierrez L Eliza M Berstein LM. Macrophages, estrogen and the microenvironment of breast cancer. J Steroid Biochem Mol Biol. (1998) 67:403–11. doi: 10.1016/S0960-0760(98)00143-5 10030689

[158] Ciesielska A Kusiak A Ossowska A Grzybowska ME. Changes in the oral cavity in menopausal women-A narrative review. Int J Environ Res Public Health. (2021) 19:253. doi: 10.3390/ijerph19010253 35010513 PMC8750983

[159] Bouma HR Bootsma H van Nimwegen JF Haacke EA Spijkervet FK Vissink A. Aging and immunopathology in primary sjögren’s syndrome. Curr Aging Sci. (2015) 8:202–13. doi: 10.2174/1874609808666150727112826 26212053

[160] Hall JE. Endocrinology of the menopause. Endocrinol Metab Clinics North America. (2015) 44:485–96. doi: 10.1016/j.ecl.2015.05.010 PMC6983294 26316238

[161] Mian AH Yang DY Kohler TS. Current management and controversies surrounding andropause. Urologic Clinics North America. (2022) 49:583–92. doi: 10.1016/j.ucl.2022.07.003 36309415

[162] Gomez CR Nomellini V Kovacs EJ. Sex Hormones and Immunosenescence. In: Fulop T Franceschi C Hirokawa K Pawelec G, editors. Handbook of Immunosenescence: Basic Understanding and Clinical Implications. Springer International Publishing, Cham (2019). p. 1457–514.

[163] Lončar-Brzak Božana Vidranski V Andabak-Rogulj A Vidović-Juras D Todorić-Laidlaw I Gabrić D. Salivary hormones and quality of life in female postmenopausal burning mouth patients-A pilot case-control study. Dentistry J. (2020) 8:111. doi: 10.3390/dj8040111 PMC7711961 33019769

[164] Agha-Hosseini F Moosavi M-S Mirzaii-Dizgah I. Salivary flow, testosterone, and femur bone mineral density in menopausal women with oral dryness feeling. Oral Surg Oral Med Oral Pathol Oral Radiol. (2013) 5:612–6. doi: 10.1016/j.oooo.2012.11.014 23433570

[165] Agha-Hosseini F Mirzaii-Dizgah I. Unstimulated saliva 17β-estradiol and xerostomia in menopause. Gynecological Endocrinol. (2012) 28:199–202. doi: 10.3109/09513590.2011.593668 21819337

[166] Pisanty S Rafaely B Polishuk W. The effect of steroid hormones on buccal mucosa of menopausal women. Oral Surg Oral Med Oral Pathol. (1975) 9:346–53. doi: 10.1016/0030-4220(75)90418-1 1058422

[167] Vittek J Kirsch S Rappaport SC Bergman M Southren AL. Salivary concentrations of steroid hormones in males and in cycling and postmenopausal females with and without periodontitis. J Periodontal Res. (1984) 19:545–55. doi: 10.1111/j.1600-0765.1984.tb01311.x 6238154

