# Supplementary material for: Sex steroid hormones: an overlooked yet fundamental factor in oral homeostasis in humans
Source: Front Endocrinol (Lausanne). 2024 Jul 23;15:1400640. doi: 10.3389/fendo.2024.1400640 (PMC11300310; doi:10.3389/fendo.2024.1400640)
Supplement: Supplementary file 1 [file Table_1.docx]

# Appendix

**Appendix 1.** Search strategy

**Table 5.** Search query April 6^th^, 2020 on PubMed database

| Search | Query | Items found |
| --- | --- | --- |
| #7 | #6 NOT ("Animals"[Mesh] NOT "Humans"[Mesh]) | 10962 |
| #6 | #2 AND #3 | 22252 |
| #5 | #4 NOT ("Animals"[Mesh] NOT "Humans"[Mesh]) | 439 |
| #4 | #1 AND #3 | 684 |
| #3 | "Gonadal Steroid Hormones"[Mesh] OR "Estrogens" [Pharmacological Action] OR sex steroid*[tiab] OR sex hormone*[tiab] OR estradiol[tiab] OR oestradiol[tiab] OR estrogen*[tiab] OR oestrogen*[tiab] OR progesteron*[tiab] OR testosteron*[tiab] OR dihydrotestosteron*[tiab] OR 5-alpha-DHT[tiab] OR 5 alpha-Dihydrotestosteron*[tiab] | 406578 |
| #2 | "Oral Health"[Mesh] OR "Mouth"[Mesh] OR "Jaw"[Mesh] OR "Dental Caries"[Mesh] OR "Alveolar Bone Loss"[Mesh] OR "Periapical Tissue"[Mesh] OR "Gingivitis"[Mesh] OR oral health[tiab] OR mouth[tiab] OR oral cavity[tiab] OR gland*[tiab] OR tongue*[tiab] OR dentition*[tiab] OR periodont*[tiab] OR palate*[tiab] OR tooth[tiab] OR teeth[tiab] OR mucosa[tiab] OR gingiv*[tiab] OR caries[tiab] OR carious[tiab] OR decay[tiab] OR alveolar[tiab] OR periapical[tiab] OR jaw[tiab] OR jaws[tiab] OR maxilla*[tiab] | 1157802 |
| #1 | "Saliva"[Mesh] OR saliva*[tiab] | 107878 |

**Table 6.** Search query October 4^th^, 2023 on PubMed database

| Search | Query | Items found |
| --- | --- | --- |
| #4 | #3 NOT ("Animals"[Mesh] NOT "Humans"[Mesh]) NOT (rat[ti] OR rats[ti] OR mice[ti] OR animal[ti] OR cancer*[ti] OR therap*[ti] OR treat*[ti] OR diagnos*[ti] OR puber*[ti] OR menstrua*[ti] OR pregnan*[ti] OR menopau*[ti] OR andropau*[ti] OR microbiom*[ti] OR biofilm[ti]) | 9,941 |
| #3 | #1 AND #2 | 27,853 |
| #2 | "Gonadal Steroid Hormones"[Mesh] OR "Estrogens" [Pharmacological Action] OR "sex steroid*"[tiab] OR "sex hormone*"[tiab] OR estradiol[tiab] OR oestradiol[tiab] OR estrogen*[tiab] OR oestrogen*[tiab] OR progesteron*[tiab] OR testosteron*[tiab] OR dihydrotestosteron*[tiab] OR "5-alpha-DHT"[tiab] OR "5 alpha-Dihydrotestosteron*"[tiab] OR "steroid hormon*"[tiab] OR "gonadal hormon*"[tiab] OR androgen*[tiab] OR progestogen*[tiab] | 508,057 |
| #1 | "Oral Health"[Mesh] OR "Mouth"[Mesh] OR "Jaw"[Mesh] OR "Dental Caries"[Mesh] OR "Alveolar Bone Loss"[Mesh] OR "Periapical Tissue"[Mesh] OR "Gingivitis"[Mesh] OR "oral health"[tiab] OR "dental health"[tiab] OR "gum health"[tiab] OR mouth[tiab] OR "oral cavity"[tiab] OR gland*[tiab] OR tongue*[tiab] OR dentition*[tiab] OR periodont*[tiab] OR palate*[tiab] OR tooth[tiab] OR teeth[tiab] OR mucosa[tiab] OR gingiv*[tiab] OR caries[tiab] OR carious[tiab] OR decay[tiab] OR alveolar[tiab] OR periapical[tiab] OR jaw[tiab] OR jaws[tiab] OR maxilla*[tiab] OR "oral soft tissue*"[tiab] OR "dental tissue*"[tiab] OR "oral epithelium"[tiab] OR "mucous membrane*"[tiab] OR orofacial[tiab] | 1,351,579 |

**Table 7.** Search query October 4^th^, 2023 on EMBASE database

| Search | Query | Items found |
| --- | --- | --- |
| #4 | #3 NOT ('Animals'/exp NOT 'Humans'/exp) NOT (rat OR rats OR mice OR animal OR cancer* OR therap* OR treat* OR diagnos* OR puber* OR menstrua* OR pregnan* OR menopau* OR andropau* OR microbiom* OR biofilm):ti NOT ('chapter'/it OR 'conference abstract'/it OR 'conference review'/it OR 'editorial'/it) | 12,781 |
| #3 | #1 AND #2 | 39,166 |
| #2 | 'sex hormone'/de OR 'androgen'/exp OR 'estrogen'/exp OR 'gestagen'/exp OR (‘sex steroid*’ OR ‘sex hormone*’ OR estradiol OR oestradiol OR estrogen* OR oestrogen* OR progesteron* OR testosteron* OR dihydrotestosteron* OR ‘5-alpha-DHT’ OR ‘5 alpha-Dihydrotestosteron*’ OR ‘steroid hormon*’ OR ‘gonadal hormon*’ OR androgen* OR progestogen*):ti,ab,kw | 777,552 |
| #1 | 'dental health'/exp OR 'mouth'/exp OR 'jaw'/exp OR 'dental caries'/exp OR 'alveolar bone loss'/exp OR 'periodontium'/exp OR 'gingivitis'/exp OR (‘oral health’ OR ‘dental health’ OR ‘gum health’ OR mouth OR ‘oral cavity’ OR gland* OR tongue* OR dentition* OR periodont* OR palate* OR tooth OR teeth OR mucosa OR gingiv* OR caries OR carious OR decay OR alveolar OR periapical OR jaw OR jaws OR maxilla* OR ‘oral soft tissue*’ OR ‘dental tissue*’ OR ‘oral epithelium’ OR ‘mucous membrane*’ OR orofacial):ti,ab,kw | 1,637,151 |
